# Role of monodentate formate in product selectivity for CO_2_ hydrogenation on Pd-based alloy catalysts

**DOI:** 10.1039/d5fd00125k

**Published:** 2026-02-03

**Authors:** Igor Kowalec, Herzain I. Rivera-Arrieta, Zhongwei Lu, Lucas Foppa, Matthias Scheffler, C. Richard A. Catlow, Andrew J. Logsdail

**Affiliations:** a Cardiff Catalysis Institute, School of Chemistry, Cardiff University Park Place Cardiff CF10 3AT Wales UK KowalecI@cardiff.ac.uk LogsdailA@cardiff.ac.uk; b The NOMAD Laboratory at the Fritz Haber Institute of the Max Planck Society Faradayweg 4-6 Berlin 14195 Germany; c Kathleen Lonsdale Materials Chemistry, Department of Chemistry, University College London London WC1H 0AJ UK; d UK Catalysis Hub, Research Complex at Harwell, Rutherford Appleton Laboratory Harwell Oxon OX11 0FA UK; e Molecular Simulations from First Principles e.V. Berlin Germany

## Abstract

The hydrogenation of CO_2_ to methanol is a key reaction for sustainable fuel synthesis. A crucial aspect of the catalytic mechanism is the role of monodentate formate (HCOO^m^*) in the initial steps of CO_2_ hydrogenation on Pd-based alloy catalysts, which we have investigated using density functional theory (DFT) together with subgroup discovery (SGD) analysis. The reactivity and stability of CO_2_ and formate species are examined on monometallic Pd, Cu, Zn surfaces and alloyed CuPd and PdZn surfaces. PdZn surfaces show low activation energy barriers for CO_2_ hydrogenation and, combined with weak CO_2_^*δ*−^ adsorption energy, this suggests that an Eley–Rideal mechanism may dominate over Langmuir–Hinshelwood pathways. The adsorption energy of the monodentate formate intermediate is found to correlate significantly with the activation energy of CO_2_ hydrogenation across all investigated facets, where stronger adsorption yields lower activation energy, enabling its use as a predictive descriptor. To determine possible new catalytic materials, a dataset of 49 Pd-based single-atom alloy (SAA) surfaces is screened using SGD, identifying key electronic parameters, the dopant and site electron affinity, as drivers of strong HCOO^m^* adsorption. The obtained subgroup discovery rules highlight Mo, Nb, and W as promising earth-abundant dopants for Pd-based catalysts, further confirmed by DFT calculations. These findings offer a mechanistic rationale for catalyst design and demonstrate the utility of AI-guided screening in identifying efficient, sustainable alloy compositions to be used as catalysts for CO_2_ conversion.

## Abstract 2

The initial hydrogenation of CO_2_ to formate is investigated on Pd-based alloy catalysts using density functional theory together with subgroup discovery analysis.

## Graphical abstract

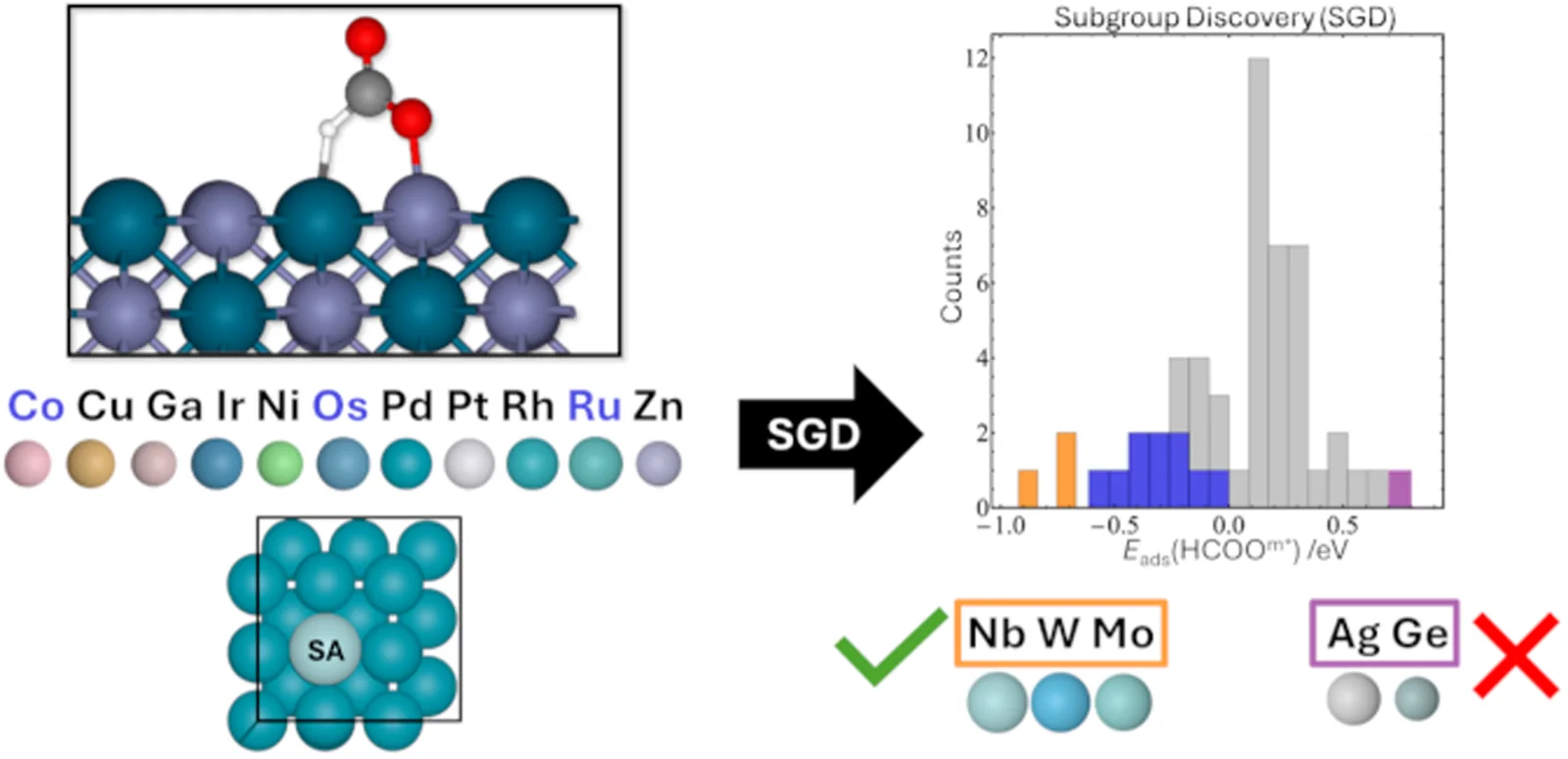

## 1 Introduction

The hydrogenation of CO_2_ to produce methanol is a promising approach to mitigate industrial emissions of a greenhouse gas while simultaneously synthesising “green” fuel and a chemical substrate of considerable societal value. The commercially-leading catalyst for hydrogenation of CO_2_ is a Cu/ZnO/Al_2_O_3_ (CZA) material, with both the ZnO support and the metallic Cu playing key roles in the catalytic mechanism.^1^ CZA is a very efficient catalyst but must operate under high temperature and pressure conditions, and is prone to deactivation in the presence of the water produced in the reaction.^2^ Recently, Pd-based supported catalysts have been investigated for selective hydrogenation of CO_2_ to methanol, showing significantly improved water tolerance compared to the prior catalysts and desirable selectivity to only C_1_ products,^3,4^ though with undesirable methane produced as well as the desired methanol product.

To explain the reactivity of Cu and Pd catalysts for CO_2_ hydrogenation, experimental studies have been beneficially complemented by computational simulations using density functional theory (DFT).^5–9^ The commonly modelled low energy (111) surfaces of face-centred-cubic (FCC) Cu and Pd catalysts show high energy barriers in reactions involving carbon dioxide and formate, an important intermediate in methanol synthesis, with less stable (100) and (110) facets likely to be more reactive and catalytically relevant.^7,8,10^ On Cu and Pd surfaces, CO_2_ has been shown to adsorb in either physisorbed (yielding CO_2_) or chemisorbed (yielding CO_2_^*δ*−^) configurations.^7,8^ Physisorbed CO_2_ orients parallel to the surface at a distance of over 3 Å and no chemical bond forms between the catalyst and the adsorbate, which means the interaction is only marginally exothermic.^7,8^ In contrast, the chemisorbed CO_2_^*δ*−^ assumes a bidentate geometry with the formation of C–metal and O–metal bonds, and electron density transfers from the metal surface to the π* orbital of the carbon atom, resulting in a distorted geometry with a reduction of the O–C–O bond angle (to 140°) that helps to stabilise the negative charge (^*δ*−^).^7,8^ On Zn (0001) surfaces, only the weakly bonded physisorbed CO_2_ species forms, with chemisorption on Zn surfaces uncommon in the absence of surface oxygen or a high electrostatic potential.^11–15^

The activity of Pd surfaces for CO_2_ hydrogenation is limited by the highly endothermic steps in the formation of the formate intermediate, with subsequent hydrogenation of formate to either methane or methanol having lower activation energies.^8,9^ The initial stages of electrocatalytic CO_2_ hydrogenation on Pd-based catalysts have been shown to involve a monodentate formate (HCOO^m^) intermediate, with only a single metal–oxygen bond between the catalyst and the molecule.^16^ The so-called formate pathway, where the carbon in CO_2_ is hydrogenated to yield the HCOO intermediate, has been linked to an increased selectivity towards methanol in catalytic systems that involve Cu, Pd and Zn metals.^7,17–19^

Alloying Pd catalysts with Zn has been shown to facilitate tuning of the reaction selectivity, with no methane observed in thermal CO_2_ hydrogenation at ambient pressure and temperatures up to 500 K.^20,21^ X-ray and neutron powder diffraction measurements were used to describe precisely the structure of the PdZn catalyst, identifying prominence of a tetragonal β_1_-PdZn alloy phase.^22^ The structure of the PdZn nanoparticle catalysts was studied using various microscopy methods and identified tetragonal crystals with greatest abundance of (101) and (110) facets.^23–26^ The 1 : 1 PdZn alloy was identified, showing features of a perfectly ordered material with a pattern of alternating stripes of Pd and Zn when viewed in the *xy*-plane.^23–27^ Theoretical studies of the PdZn alloy at varying composition confirmed the 1 : 1 ratio as most energetically favourable, and identified the most stable structure as body-centred tetragonal, in agreement with experiment.^28^

An alternative catalyst composition of Pd alloyed with Cu has been shown to also enhance CO_2_ hydrogenation reactivity, relative to monometallic systems, with geometry^29^ and electronic^30^ effects from the CuPd alloying important in defining the selectivity towards C_1_ and C_2_ products. Alloying Pd with Cu enhances the thermally-driven CO_2_ hydrogenation reaction, by means of a synergetic effect of hydrogen spill-over from Pd onto the Cu active sites,^31^ as corroborated by multiple studies.^27,30,32–34^ Challenges remain, however, with respect to understanding the reaction mechanism on these surfaces, particularly with respect to the stability of the formate species and the most prominent reaction mechanism.^35^

The mechanism of CO_2_ hydrogenation to methanol on these supported alloy nanoparticle catalysts is demonstrably linked to the formate intermediate,^35^ and application of novel computational tools to probe the chemical reactivity of this key intermediate can facilitate new understanding and rational catalyst design. Simulations of homogeneous alloys can be coupled with dilute-limit representations of the catalyst, *i.e.*, single-atom alloy (SAA) catalysts, to understand concentration effects; furthermore, SAAs offer a platform for high-throughput screening of catalysts, for tuning of the active sites, exploring structure–activity relationships, and improving reactivity with small metal loadings.^34,36–38^ Theoretical approaches have successfully explained the chemical phenomena occurring on SAA catalysts, such as enhancement or creation of new active sites.^37–41^ Additionally, the surface structure of SAA catalysts is typically constrained to that of the host material, making the modelling of SAA surfaces more accessible than more disordered alloy systems, as the number of surface configurations are greatly reduced; the consequence is more efficient theoretical probing of catalytic materials can be performed on a quantitative basis.^38^ Thus, SAA catalysts offer significant opportunities to strengthen understanding of reaction mechanisms, and to tailor active sites to improve efficacy for CO_2_ hydrogenation.^42^

Here, we present an extensive study of the stability and reactivity of CO_2_ and formate species over important surfaces for Pd-based catalysts with the aim of elucidating reaction details and progressing catalyst design. We apply density functional theory (DFT) to calculate the stability and reactivity of CO_2_ and hydrogen on homogeneously alloyed body-centred tetragonal (BCT) PdZn (101) and (110) surfaces, and a body-centred cubic (BCC) CuPd (110) surface. The hydrogenation of CO_2_ to produce formate is explicitly considered, and the performance of alloys is referenced against their monometallic counterparts (Pd, Zn, and Cu). We build on our mechanistic results by surveying the stability of formate on SAA surfaces of Pd (100), (110), (111), and (211) doped with Co, Cu, Ga, Ir, Ni, Os, Pt, Rh, Ru or Zn. The SAA surfaces have been described using 13 descriptive parameters intrinsic to the materials, resulting in a complex dataset that warrants the use of Artificial Intelligence (AI) to identify the most chemically relevant information. The method applied herein is subgroup discovery (SGD), a focused AI approach devoted to finding subsets or subgroups displaying exceptional patterns within a data set.^43^ SGD is a powerful and versatile tool that can be applied to both scientific and non-scientific problems where the target property of interest is well defined either numerically or categorically. Through the combined use of DFT and SGD, we are able to highlight and confirm novel and earth-abundant transition metal alloy candidates with potential to increase the efficacy of Pd-based catalysts for CO_2_ hydrogenation.

## 2 Computational methods

### 2.1 Electronic structure calculations

The Fritz Haber Institute *ab initio* materials simulations (FHI-aims) software package has been used for full potential all-electron DFT calculations, with the Pythonic Atomic Simulation Environment (ASE) used for management of calculation geometries.^44,45^ The default convergence criteria within FHI-aims for self-consistent field (SCF) calculations were used, *i.e.*, the changes between the current and previous SCF iterations in charge density, sum of eigenvalues, and total energy were below *N* × 1.67 × 10^−5^ e a_0_^−3^, 10^−3^ eV, and 10^−6^ eV, respectively, where *N* is the number of atoms in the model. Scalar relativistic treatment of kinetic energy for all elements was achieved by the atomic zero-order regular approximation (ZORA),^46^ and a Gaussian-type broadening with width of 0.01 eV was applied to the occupation of electronic states. The accurate semi-local Bayesian error estimation density functional (mBEEF)^47^ exchange–correlation density functional (DF), as implemented in the LibXC DF library,^48^ has been previously identified as a suitable choice for modelling alloy Cu, Pd and Zn surfaces,^10^ and this DF has been applied throughout. A reciprocal space sampling of one *k*-point per (0.018 × 2π) Å^−1^, and a default “light” basis set (version: 2020), have been used for geometry optimisations, providing structural accuracy and electronic accuracy in Mulliken charge population analysis.^10,44,49^ Spin-paired calculations were used in periodic calculations; gas-phase adsorbate structures were calculated with both spin-paired and spin-unpaired configurations, and the energy of the more stable system was considered for reference to periodic calculations. For geometry optimisations, convergence was deemed complete when forces on all unconstrained atoms were less than 0.01 eV Å^−1^; a force correction term was applied for geometry optimisations also, which suppresses the strong fluctuations in the Hellmann–Feynman forces when the electron densities are loosely converged and results in speed up of ∼30%, without compromising calculation accuracy.^42^

Monometallic surfaces were constructed from face-centred cubic (FCC) bulk unit cells of Cu and Pd, and a hexagonal close-packed (HCP) unit cell of Zn, based on the outcomes of the work by Kabalan *et al.*^10^ Supercells of the surfaces were created for Pd (111), (100), and (110); Cu (111), (100), and (110); and Zn (0001), with dimensions of (3 × 3 × 7) unit cells and a vacuum layer of 40 Å added in the *z*-direction. The bottom three atomic layers were constrained to their bulk positions, with the 4 top surface layers remaining unconstrained; and, due to the one-sided nature of the slab models, a dipole-correction was used in all calculations. The alloy surfaces were prepared similarly to the monometallic surfaces; however, as BCT and BCC primitive cells contained two atoms, (2 × 3 × 7) supercells were used. For the body-centred tetragonal (BCT) PdZn (101) and (110) surfaces, the bimetallic alloy ordering was matched to experimental studies by Peterson *et al.* and Nowicka *et al.,*^22,26^ with the greater stability of PdZn (110) confirmed by our calculations. The ordering of the body-centred cubic (BCC) CuPd (110) surface was based on a study by Yamauchi *et al.*^50^ Following the naming convention of Crawley *et al.* the investigated ordered (o-) alloy catalysts could be described as body-centred tetragonal (BCT) o-Pd_0.5_Zn_0.5_ and body-centred cubic (BCC) o-Cu_0.5_Pd_0.5_.^51^ Here, we refer to these alloys as PdZn and CuPd, respectively, with crystal structure, composition and ordering implied for simplicity. All prepared facets are presented in Fig. S1 in the SI.

To study the catalysed surface reactions, the adsorption energy (*E*_ads_) measuring the interaction between a surface and adsorbate is deduced from comparison of energies of optimised gas-phase adsorbate (*E*_adsorbate_), pristine surface (*E*_pristine_), and the optimised combined system (*E*_slab_):*E*_ads_ = *E*_slab_ − (*E*_adsorbate_ + *E*_pristine_)For the formate species, the reference *E*_adsorbate_ is instead taken from ½H_2_ and CO_2_ to avoid referencing against the unstable gas-phase formate radical. The definition used is such that negative *E*_ads_ indicates an exothermic interaction. The *E*_ads_ of formate species on the catalyst surface (*) is considered in monodentate (HCOO^m^*) or bidentate (HCOO^b^*) form, where ^m^ and ^b^ superscripts denote mono- or bi-dentate configurations; carbon dioxide is considered either as physisorbed (CO_2_*) or chemisorbed (CO_2_^*δ*−^*); and hydrogen as dissociatively adsorbed only (H*), where * indicates a surface-bound species in all cases. To rationalise how the alloying of Pd, Cu, and Zn changes the reaction profile for CO_2_ hydrogenation to formate, models were constructed for the hydrogenation of physisorbed and chemisorbed CO_2_ to HCOO^m^* and HCOO^b^* species. The lowest lying transition state (TS) is reported in each case, as described in more detail in Section S3 of the SI. The final product state taken as an energy reference corresponds to the strongly surface-bound HCOO^b^* intermediate. For the calculation of kinetic barriers, the climbing-image MLNEB method has been used to identify saddle points and minimum energy paths (MEP).^52,53^ In line with our previous work, a spring constant of 0.05 eV Å^−1^ has been used throughout; the convergence criteria is for forces on all unconstrained atoms to be below 0.05 eV Å^−1^, with energy uncertainty below 0.03 eV.^8^

For the single atom alloy (SAA) catalysts, calculations of Pd (111), (100), (110), and (211) (Fig. S2 in the SI) surfaces were considered with a single atom in the top atomic layer substituted by one of the following elements: Co, Cu, Ga, Ir, Ni, Os, Pt, Rh, Ru and Zn. The structures were all subjected to geometry relaxation,^54^ with the self-consistent field (SCF) parameters consistent with the above details except for structures containing Co and Ni, where a spin-unpaired calculation was chosen, with the default initial magnetic moment set to 0.01 so as to allow dynamic spin convergence; for these cases, the calculations also required the occupation broadening was increased 0.1 eV, the mixer was explicitly set to Pulay, and the Kerker preconditioner was disabled, to aid SCF convergence. For studies of the SAA catalysts, the initial structure of monodentate formate species were adapted from chemisorbed CO_2_ geometries on the Pd SAA facets,^42^ and refined with the MACE-MP forcefield with a pre-trained “small” model and a van der Waals correction.^55–57^ Full details of the calculation settings are provided in the SI.

### 2.2 Subgroup discovery

From the structures optimised with DFT, a set of basic candidate parameters characterising the (clean) adsorption sites and the formate adsorption energy values were obtained. This data served as inputs for the SGD approach. The dataset constitutes 49 symmetrically inequivalent monodentate formate structures adsorbed on the surfaces of SAAs. The goal of the SGD analysis is to identify the key parameters that describe the adsorption properties of monodentate formate. A detailed description of SGD can be found in the work of S. Wrobel,^43^ with contextual examples provided by B. R. Goldsmith *et al.*^58^ and Foppa *et al.*^59^ Briefly, the SGD algorithm identifies descriptions of subsets of data (subgroups) displaying outstanding distributions of a given target of interest, in our case the adsorption energy of HCOO^m^*. The SGD descriptions or rules are typically inequalities constraining the value of key basic parameters, selected from all the offered candidate parameters. The rules provided by SGD can be used to identify new surface sites with the desired adsorption properties. The SG identification is based on the maximization of a quality function *Q*(*S*, *P*), which ensures a balance between the size of the SG and its utility, *e.g.*, the preference for low or high values of the target of interest. Here, we utilize the negative mean shift to identify SGs of surface sites with low adsorption energy of HCOO^m^*.

The 13 candidate descriptive parameters considered for SGD in this work correspond to two classes: SA and surface adsorption site. These parameters are chosen to comprise the SAA surfaces’ electronic and geometric properties. The SA parameters are the free-atom Pauling electronegativity (PE_SA_), the electron affinity (EA_SA_), the ionization potential (IP_SA_), and the radii of the s-, p-, d-, and valence orbitals (*r*_s-SA_, *r*_p-SA_, *r*_d-SA_, *r*_val-SA_). Surface sites are described using PE_site_, IP_site_, and EA_site_, where the subscript “site” implies the average of these properties over all the atoms in the adsorption ensemble, *i.e.* nearest neighbouring metal atoms. The geometry of the adsorption site is included through the coordination number (CN), the generalized CN (gen-CN), and the number of atoms in the adsorption site (*N*_site_). The technical details for the SGD studies are provided in the Section S5 in the SI.

## 3 Results and discussion

### 3.1 CO_2_ adsorption on metallic and 1 : 1 alloy surfaces

**Fig. 1:**
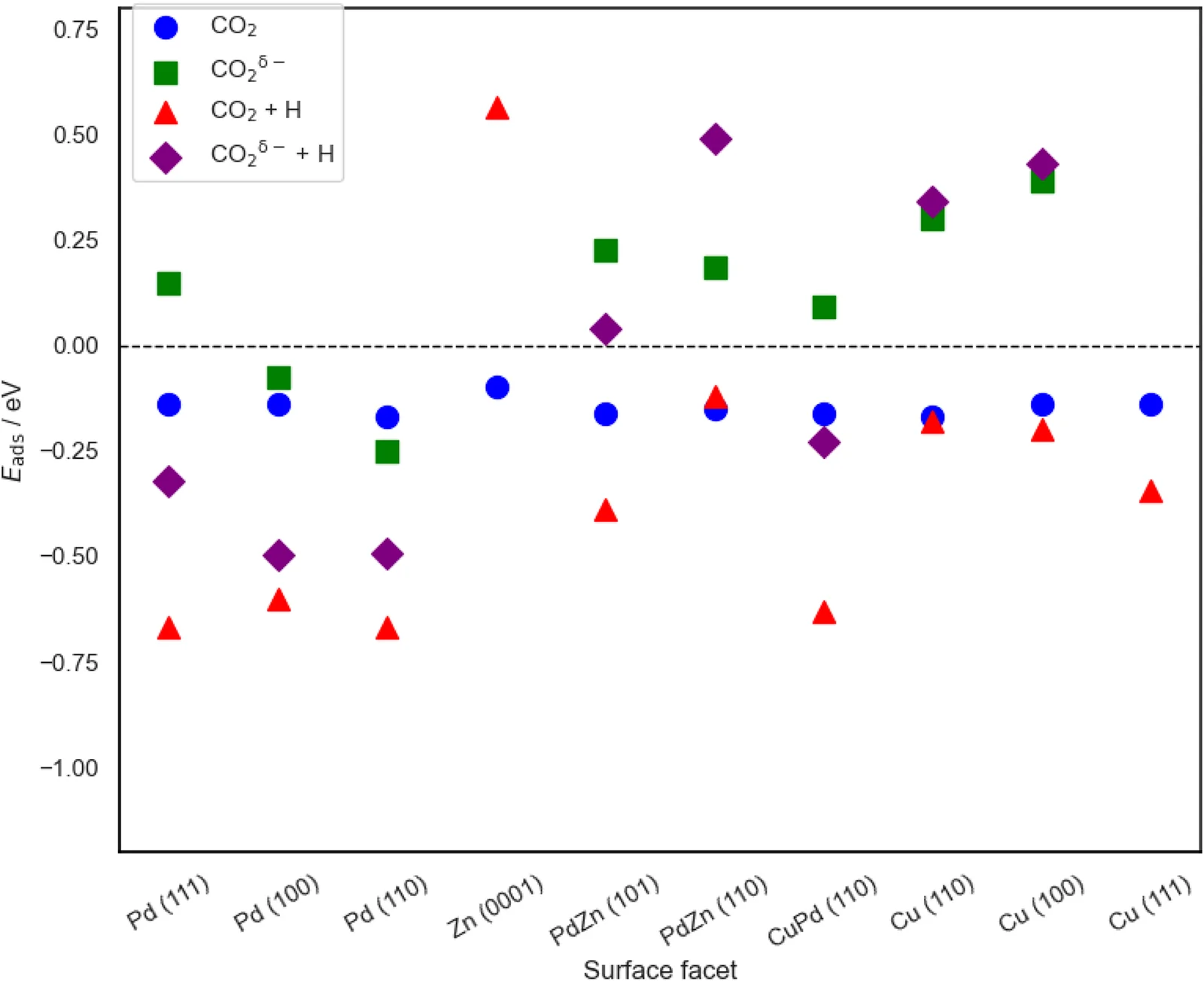
*E*_ads_ of CO_2_ and CO_2_^*δ*−^ in units of eV, with and without co-adsorbed H in the most favourable adsorption position, across Pd (111), (100), (110), Zn (0001), Cu (111), (100), (110), CuPd (110) and PdZn (101) and (110) surfaces. No metastable structures of CO_2_^*δ*−^ were found on Cu (111) and Zn (0001) surfaces. The numerical data is available in Table S4 in the SI.

To gain insight into the interaction of the key CO_2_ hydrogenation reaction substrates with the metallic Cu, Pd, Zn and CuPd and PdZn alloy surfaces, CO_2_ adsorption and co-adsorption with H was investigated on (111), (100), (110) surfaces of Cu and Pd, the (0001) Zn facet, CuPd (110), and (110) and (101) surfaces of PdZn. The most stable calculated adsorption energy, *E*_ads_, for CO_2_ on monometallic and alloy surfaces are given in Fig. 1. The physisorbed CO_2_ species has very similar stability on all investigated surfaces, with *E*_ads_ of between −0.17 and −0.10 eV; thus, the reactivity of CO_2_ on specific surfaces is difficult to deduce based on these interactions. In contrast, the stability of chemisorbed CO_2_^*δ*−^ is more varied and carries more information about reactivity.

**Fig. 2:**
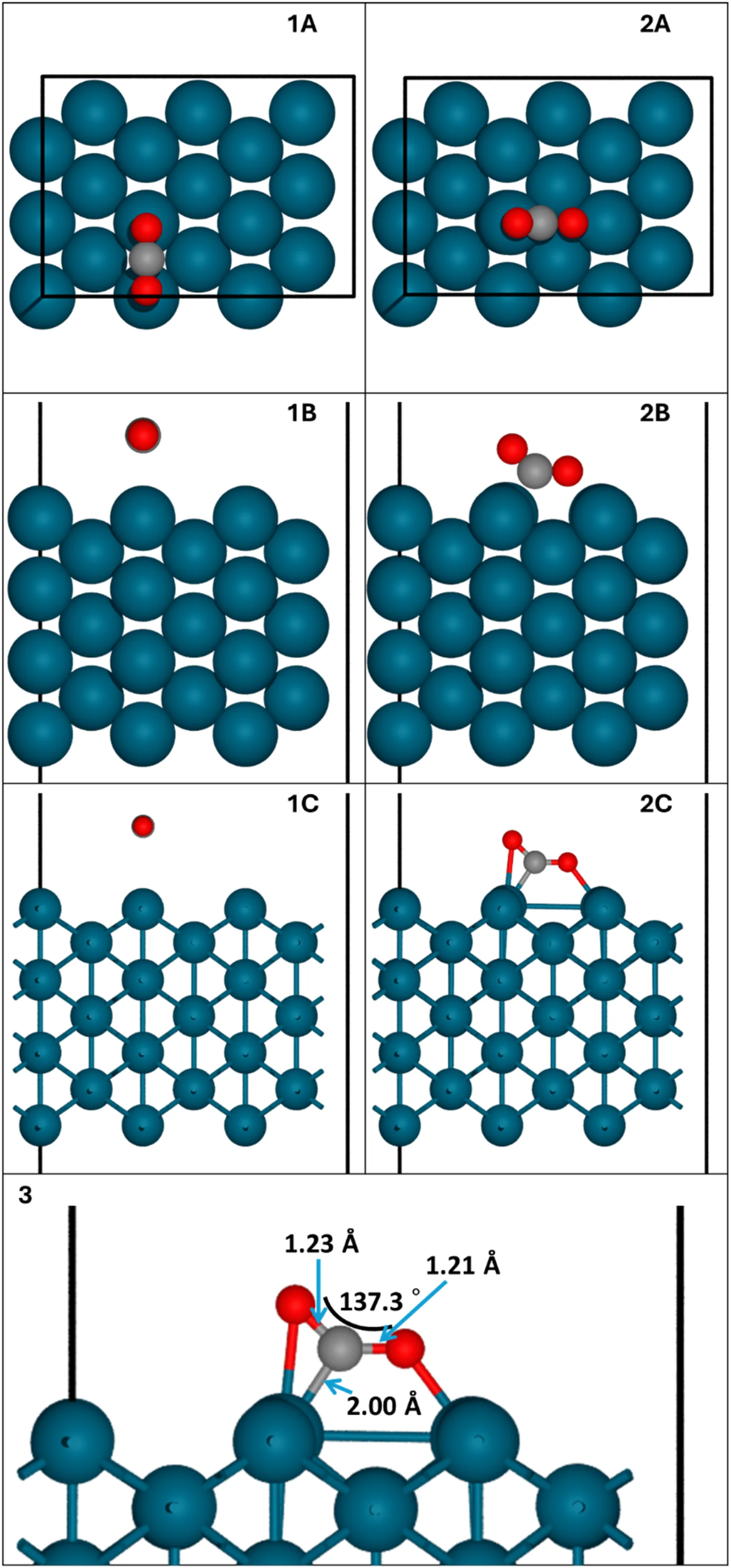
The CO_2_ adsorption geometries on Pd (110), with subfigures 1, and 2 corresponding to the CO_2_ physisorbed along the short-bridge site and CO_2_^*δ*−^ along the long-bridge site, respectively. The view is along the *xy*-, *xz*-, and *xz*-axis in sub figures A, B, and C, respectively. Subfigures 1C and 2C apply a ball and stick model to highlight bonding interactions. Subfigure 3 presents a magnified and annotate 2C figure. The black lines represent the boundaries of the unit cell. The grey, red, and blue spheres represent the C, O, and Pd atoms, respectively. The numerical data is available in Table S4 in the SI.

The exothermic CO_2_^*δ*−^ adsorption on the Pd (100) and (110) surfaces implies that these surfaces may chemically activate CO_2_, with the latter shown in Fig. 2, but the interaction is weak and therefore any chemisorbed state may be short-lived, matching our previous conclusions obtained using a PBE+TS method.^8^ The *E*_ads_ of chemisorbed CO_2_^*δ*−^ on the denser Cu and Pd surfaces was calculated as 0.39 eV and 0.15 eV on Cu (100) and Pd (111), respectively, and no CO_2_^*δ*−^ structure was observed on Cu (111), in line with observations of Higham *et al.*^7^ The calculated *E*_ads_ of CO_2_^*δ*−^ on Cu (110) in the current work is weaker than reported by Higham *et al.*, which is attributed to differences in calculations settings; we note that the mBEEF exchange–correlation density functional used here has proven greater accuracy for both material and adsorption processes in recent testing and other works.^8,10,60^

**Fig. 3:**
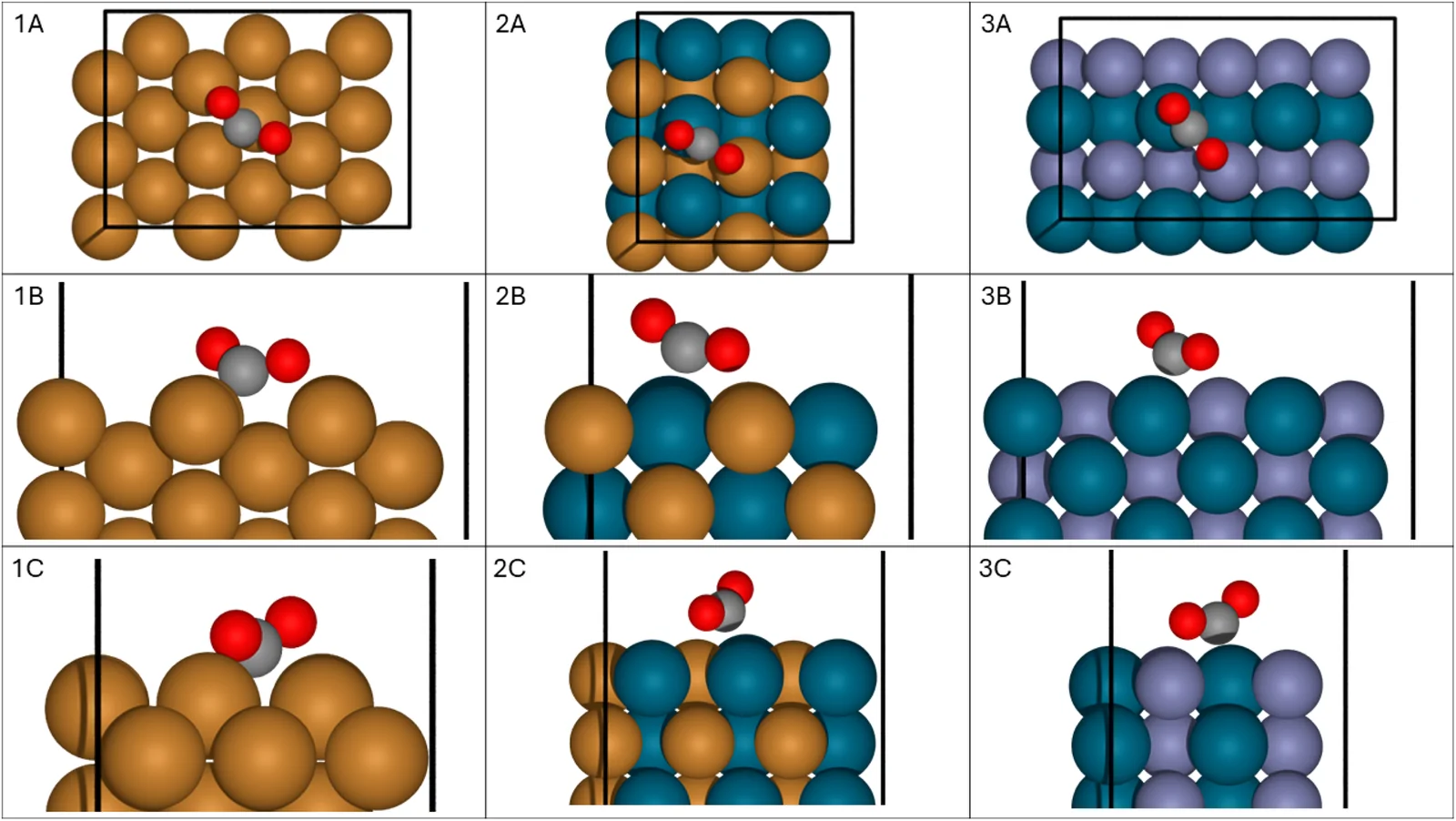
The CO_2_^*δ*−^ adsorption geometries on (1) Cu (110), (2) CuPd (110) and (3) PdZn (110). The view is along the *xy*-, *yz*-, and *xz*-axis in A, B, and C, respectively. The black lines represent the boundaries of the unit cell. The grey, brown, red, blue, and purple spheres represent the C, Cu, O, Pd, and Zn atoms, respectively.

The *E*_ads_ of CO_2_^*δ*−^ on PdZn (101) and (110) is 0.22 eV and 0.18 eV, respectively, which is weaker than the strongest adsorption on pure Pd (111). The most stable CO_2_^*δ*−^ geometries are on the Cu (110), Pd (110), CuPd (110), and PdZn (110) facets for the different metal surfaces, with an *E*_ads_ of 0.30 eV, −0.25 eV, 0.18 eV and 0.09 eV, respectively. The adsorbed structures are shown in Fig. 2 and 3, with additional structural information available in Table S4 in the SI.

The endothermic CO_2_^*δ*−^ adsorption on Cu (100), (110), Pd (111), CuPd (110) and PdZn (101) and (110) surfaces shows that CO_2_^*δ*−^ on these surfaces is likely a transient species. For the CuPd and PdZn alloy surfaces, only a single chemisorbed structure of CO_2_^*δ*−^ is identified. On the bimetallic PdZn surfaces, the bidentate CO_2_^*δ*−^ species binds on intermetallic bridging sites, with C more strongly bound at Pd and O at Zn; the preferable geometry of CO_2_^*δ*−^ adsorbed on CuPd (110) is similar, with Pd–C and Cu–O bonds, and the monometallic adsorption sites are unstable.

Hydrogenation of adsorbed CO_2_ to produce formate requires a H* species in the vicinity of the carbon dioxide; therefore, the relative stability of the CO_2_ and CO_2_^*δ*−^ were assessed with co-adsorption of hydrogen, and the results are also provided in Fig. 1. Other than for the Pd surfaces, the initial co-adsorbed CO_2_^*δ*−^* and H* state, required for the Langmuir–Hinshelwood mechanism, is exothermic only on CuPd (110), with all other surfaces endothermic or unstable [Zn (0001); Cu (111)]. Given the endothermicity of the Langmuir–Hinshelwood reactant state, alternative mechanisms, such as the Eley–Rideal, might be playing a significant role in initial CO_2_ hydrogenation to formate on PdZn surfaces, as was previously observed for Cu surfaces.^61^

### 3.2 HCOO

**Fig. 4:**
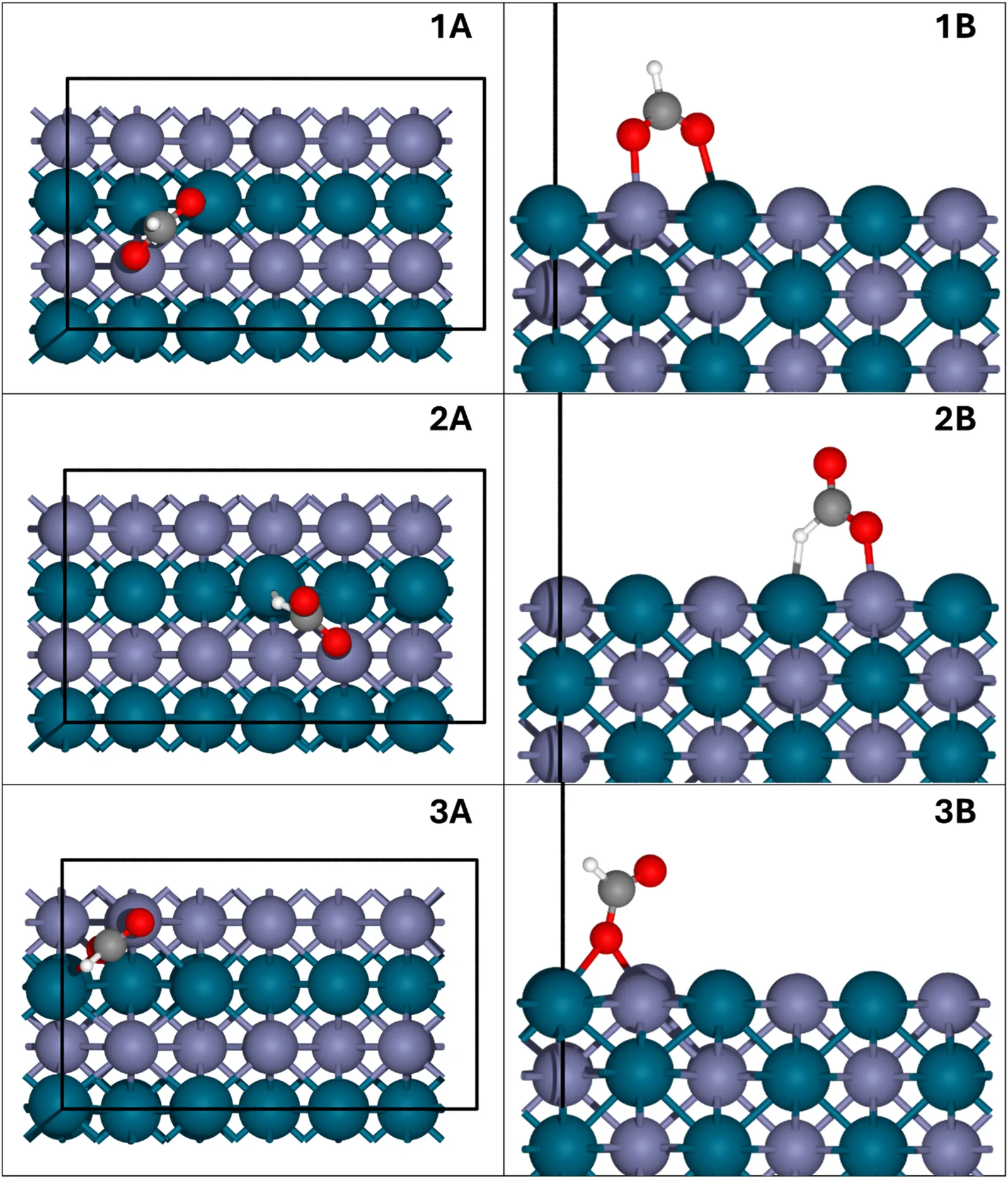
Three types of formate intermediates considered on the BCT PdZn (110) facet: bidentate (1) and monodentate with (2) and without (3) a significant hydrogen–metal interaction, in *xy*- and *yz*-view (A and B, respectively); blue, purple, red, grey and white spheres represent Pd, Zn, O, C, and H atoms, respectively; the black lines are boundaries of the simulation cell. The numerical data is available in Table S4.

Three binding modes of the HCOO* intermediate have previously been identified on Cu surfaces, which correspond to two monodentate configurations (HCOO^m^*, with and without hydrogen atom interacting with the surface) and a bidentate configuration (HCOO^b^*).^62–66^ As the FCC Cu and Pd (110) surfaces were previously highlighted as more catalytically active towards CO_2_ hydrogenation than the more stable (111) and (100) facets,^7,8^ the relevance of the formate species in CO_2_ hydrogenation was investigated by studying its stability on the Cu and Pd (110) surfaces, as well as the prominent alloy surfaces of CuPd^50^ [(110)] and PdZn^26^ [(101) and (110)]. The formate adsorbate was initially positioned 2 Å above the adsorption sites, with either both oxygen atoms, one oxygen and one hydrogen atom, or just one oxygen atom facing towards the surface, with example resulting structures on the PdZn (101) surface depicted in Fig. 4. The *E*_ads_ of the HCOO* adsorption modes on Cu (110), Pd (110), CuPd (110), PdZn (101) and PdZn (110) surfaces are shown in Table 1.

**Table 1:** *E*_ads_(HCOO) on FCC Cu and Pd (110) surfaces, BCC CuPd (110) and BCT PdZn (101) and (110) surfaces, relative to gas-phase reactant species (½H_2_ and CO_2_); ^m^* corresponds to a monodentate adsorption geometry and ^b^* corresponds to a bidentate adsorption geometry. In some cases, metastable formate configurations were identified with the H moiety directed towards the surface to form a H–M interaction (where M is a metal species), and the *E*_ads_ in these cases is given in bold. Models showing unstable adsorption are marked with dashes

	*E*_ads_(HCOO^m^*)/eV		*E*_ads_(HCOO^b^*)/eV	
**Adsorption site**	**Cu (110)**	**Pd (110)**	**Cu (110)**	**Pd (110)**
Short-bridge	−0.40, **0.09**	0.14, **0.17**	−1.08	−0.81
Long-bridge	—	—	−1.02	−0.65

**Adsorption site**	**CuPd (110)**			
Pd–Pd bridge	**0.58**		−0.34	
Pd–Cu bridge	**0.50**		−0.43	
Cu–Cu bridge	0.15		−0.50	

**Adsorption site**	**PdZn (101)**			
Pd–Zn–Zn	−0.21		−0.70	
Pd–Pd–Zn	−0.28, **0.12**		−0.64	
Pd–Zn	—		−0.82	

**Adsorption site**	**PdZn (110)**			
Pd–Zn–Zn	0.17		—	
Zn–Zn	—		−0.72, −0.75	
Pd–Zn	−0.37, **−0.03**		−0.87	
Pd–Pd	—		−0.41, −0.44	

Notably, the adsorption of monodentate HCOO (HCOO^m^*) without a H–M interaction (where M is a metal species) is endothermic on FCC Pd (110) and BCC CuPd (110) surfaces, and exothermic on the FCC Cu (110), BCT PdZn (101) and (110) surfaces. The only exothermic HCOO^m^* species with a H–M interaction, which might be produced by initial CO_2_ hydrogenation, is for the PdZn (110) surface. The bidentate geometry (HCOO^b^*) is more stable for all systems, especially on the short-bridge site of the FCC (110) surfaces of Cu and Pd, with *E*_ads_ of −1.08 eV and −0.81 eV, respectively; HCOO^b^* is most stable at the Cu–Cu bridge adsorption site of BCC CuPd (110) surface, with *E*_ads_ of −0.50 eV. HCOO^b^* is more stable on Cu (110) than on Pd (110), and the preferred adsorption on the Cu atoms of BCC CuPd (110) surface layer matches this observation: *E*_ads_ of HCOO^b^* is −0.34 eV when both oxygens are bound to Pd, and −0.43 eV and −0.50 when bound to one and two Cu atoms, respectively. In contrast, adsorption of HCOO^b^* involving multiple Pd or multiple Zn atoms on PdZn (101) and (110) facets is weaker than on the most stable bimetallic Pd–Zn bridge sites, with *E*_ads_ of −0.82 and −0.87 eV on PdZn (101) and (110), respectively, *i.e.*, similar to *E*_ads_ of HCOO^b^* on the Pd (110) surface. The most stable HCOO^b^* structure on the BCC CuPd (110) surface is significantly less stable than on the monometallic Cu (110) and Pd (110) surfaces, by 0.58 eV and 0.31 eV, respectively, suggesting that the CuPd catalyst surface is less prone than Cu to poisoning with the formate species.

Under reaction conditions, the formate species is likely to primarily bond in the bidentate configuration, due to its greater stability on each investigated metal and alloy surface. However, when a HCOO species is formed from CO_2_ and H*, the H* transfer from the surface means a non-vertical or non-bidentate position must initially form to facilitate the transfer of the surface-bound hydrogen onto the carbon; this reaction step is highly endothermic, as previously shown on Cu and Pd facets.^7,8,65^ Horizontally positioned formate species that correspond to TS structures observed in simulations on Cu^7^ and Pd^8^ surfaces are not metastable; however, the observed metastable HCOO^m^* species with an H–M interaction resembles the TS structure for HCOO formation in an alternative study of formate desorption on Cu (110), and is proposed to be an important intermediate.^65^

Mulliken charge analysis of the HCOO configurations shows that a major part of the electron density withdrawn from the surfaces is towards the oxygen atoms. The C atom has a charge that is 0.20 e higher in the HCOO^b^* and HCOO^m^* formate structure, with H–M interaction, than for the physisorbed CO_2_. The charge on the H atom in the H–M configuration of HCOO^m^* is −0.04 to −0.08 e lower (*i.e*., less positive charge, more electrons) than in HCOO^b^*. The H–M interaction may enhance an Eley–Rideal-type mechanism for the formation of formate, where physisorbed CO_2_ interacts with the surface-adsorbed H, thus bypassing the CO_2_ chemisorption necessary for the Langmuir–Hinshelwood mechanism.

**Fig. 5:**
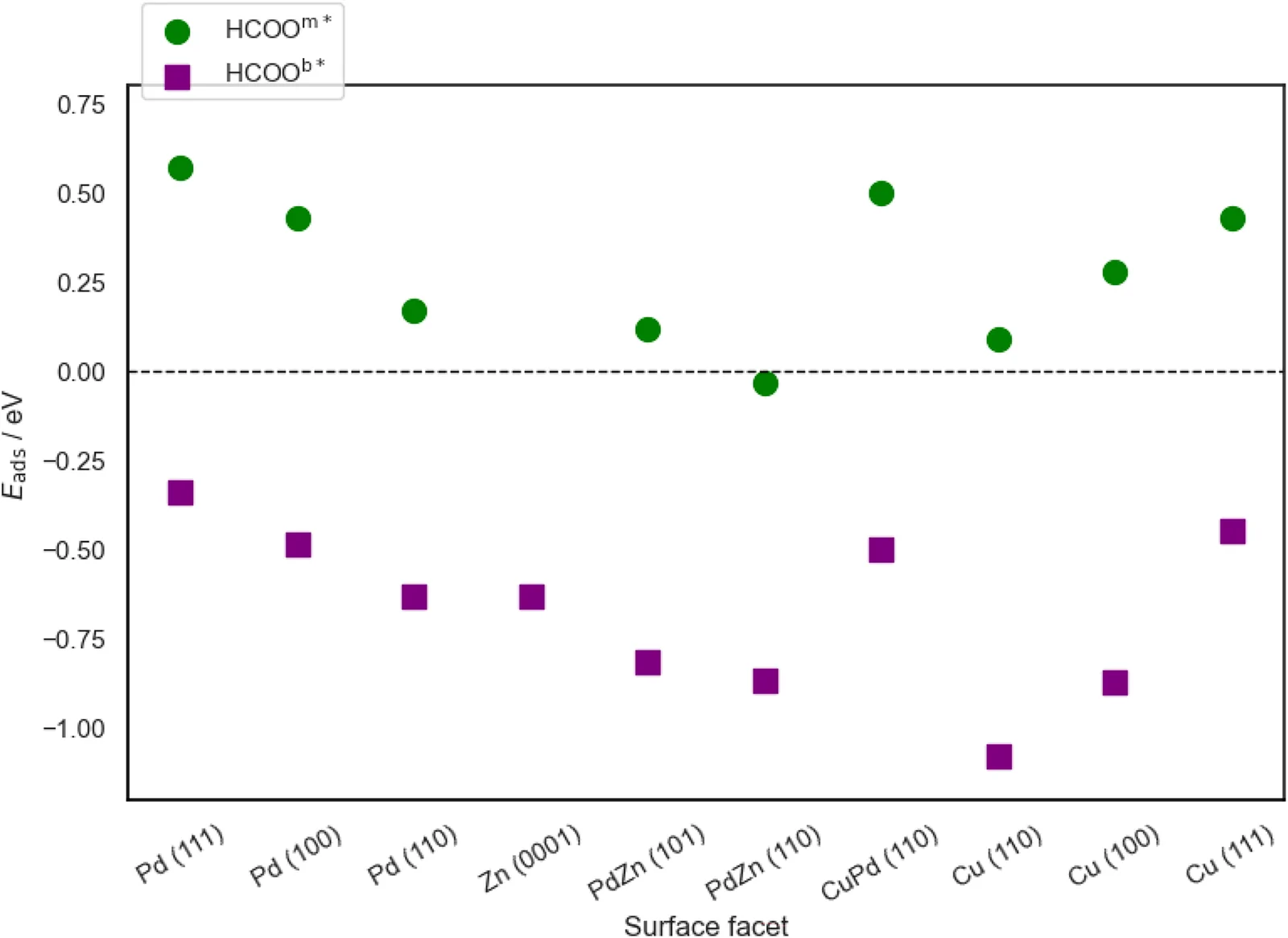
*E*_ads_ of surface-bound (*) monodentate (^m^) and bidentate (^b^) formate (HCOO) intermediates on FCC Pd (111), (100), (110), Cu (111), (100), (110), HCP Zn (0001), BCC CuPd (110), and PdZn (101) and (110) surfaces. HCOO^b^* is in the most stable conformation with both oxygen atoms bound to metal atoms, while the HCOO^m^* is bound to the surface *via* a single oxygen atom and a significant H–metal electronic interaction. No metastable HCOO^m^* structure has been identified on the Zn (0001) surface. The numerical data is available in Table S4 in the SI.

The stability of the formate adsorbates was explored further, considering the most stable HCOO^b^* and the HCOO^m^* intermediates with an H–M bond that might enable alternative reaction mechanisms. The HCOO^b^* and HCOO^m^* intermediates were modelled on the FCC Pd (111), (100), FCC Cu (111), (100), HCP Zn (0001), and the results compare to those presented in Table 1. The *E*_ads_ of the HCOO^b^* and HCOO^m^* with a H–M interaction were calculated on each surface and are shown in Fig. 5. No metastable HCOO^m^* structure has been identified on the Zn (0001) surface, similar to the inability to chemisorb CO_2_^*δ*−^.

Li *et al.* previously reported the adsorption energy of monodentate formate on Pd (111) and Pd (100) surfaces to be 0.76 eV and 0.78 eV weaker than the bidentate species,^67^ respectively, which is comparable to the energy differences of 0.91 eV calculated for both surfaces here. The only exothermic *E*_ads_(HCOO^m^*) with a H–M interaction is for the PdZn (110) surface, with a marginally negative *E*_ads_ of −0.03 eV. The recognition of the *E*_ads_(HCOO^m^*) outlier informs both further reactivity investigations and hints at potential targets for SGD analysis.

### 3.3 Reaction energetics

**Fig. 6:**
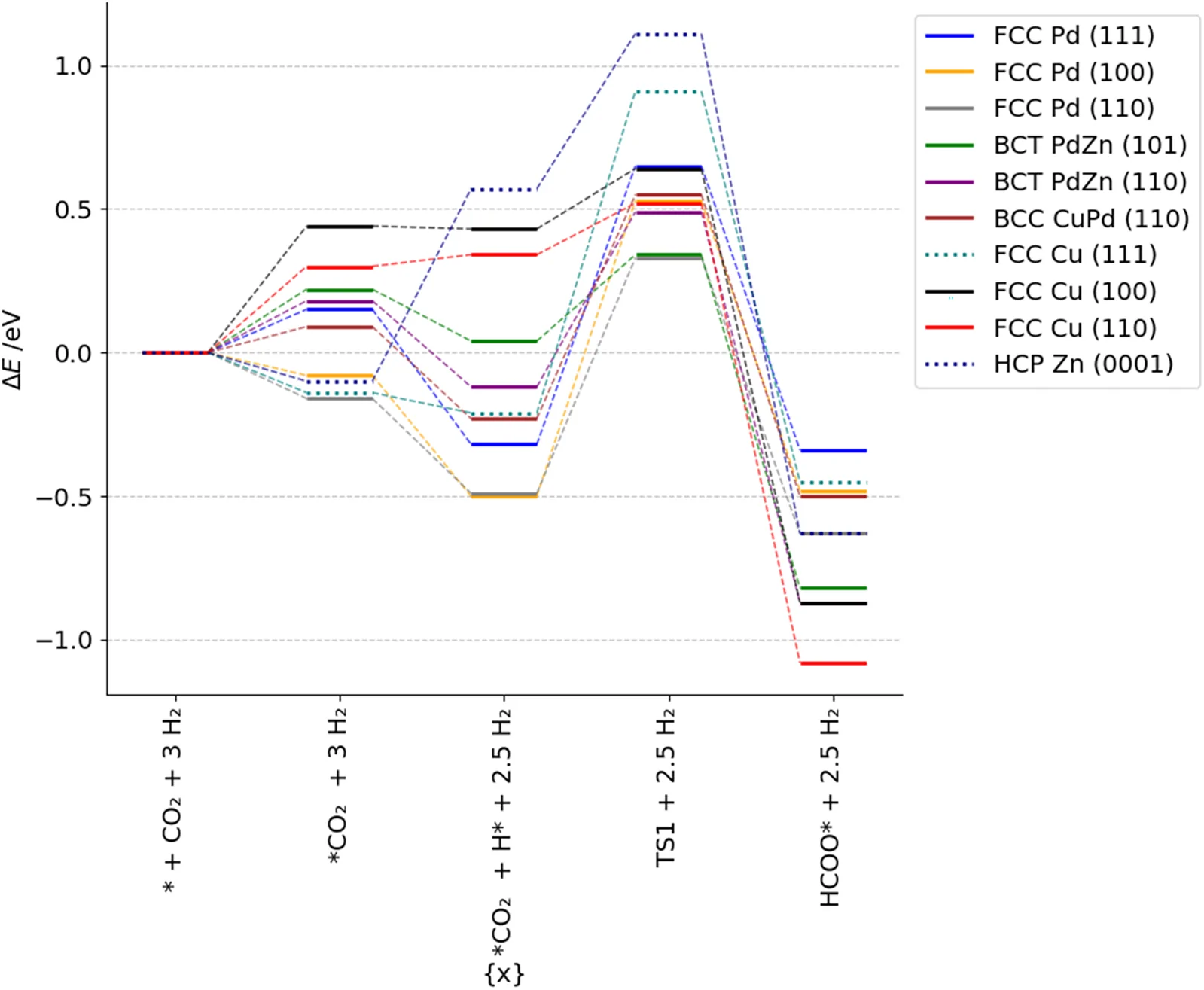
Reaction energy profiles of initial hydrogenation of CO_2_ to HCOO on FCC Cu, Pd, HCP Zn, BCT PdZn and BCC CuPd surfaces in units of eV. The preferred adsorbed CO_2_ geometry for the transition state calculation was the bent chemisorbed CO_2_^*δ*−^ (solid line). Metastable CO_2_^*δ*−^ geometry was not found on FCC Cu (111) and HCP Zn (0001), thus the physisorbed CO_2_ was used instead (dotted lines); * denotes a surface and surface-bound intermediates. The numerical data is available in Table S4 in the SI.

The mechanism of CO_2_ hydrogenation on Cu (100), (110) and (111) facets has been proposed to involve the monodentate HCOO^m^* species, from which the HCOO^b^* is formed without an energy barrier.^66^ Notably, the calculated *E*_ads_ of the TS for the initial CO_2_ hydrogenation to formate is similar to the adsorption energy of the HCOO^m^* species.^66^ An alternative mechanism, with a horizontally positioned HCOO^b^* species, including the H–M interaction and two O–M bonds, was recently reported from DFT calculations with a barrierless rotation to HCOO^b^* on Cu (100) and (110) facets.^7^ The HCOO^m^* species has been highlighted as a key intermediate in electrochemical reduction of CO_2_ on Pd surfaces, and so there is a clear need to investigate further.^16^ The activation energy of hydrogenation of physisorbed CO_2_ to formate, *E*_a_(CO_2_ + H), activation energy of CO_2_^*δ*−^ hydrogenation to formate, *E*_a_(CO_2_^*δ*−^ + H), and the reverse reaction, *i.e.*, the activation energy for decomposition of bidentate formate, *E*_a_(HCOO^b^) were calculated, and the reaction energy profile is presented in Fig. 6, and the geometric data in Table S4 in the SI.

Considering the TS structures obtained across the investigated Pd, Zn and PdZn surfaces, the carbon–metal bond (*d*_TS_(C–M)) is longer on PdZn than on Pd and the C–H distance in the TS changes significantly from 1.15–1.23 Å on Pd to 1.60–1.68 Å on PdZn surfaces, similar to the 1.63 Å for the monometallic Zn surface; the C–H distance on Cu surfaces also spans from 1.61–1.63 Å, whilst the distance on the CuPd (110) surface is intermediary to Cu and Pd (1.43 Å). The results imply that the H transfer from the metal surface to the C is most energy intensive for the Pd surface, while the CO_2_ activation and bending requires the most energy on Cu, Zn and PdZn surfaces. Further, both the HCOO^b^* and HCOO^m^* species have stronger (more exothermic) *E*_ads_ on lower coordination (and higher energy) surfaces; the HCOO^m^* species is also noted as less stable than CO_2_^*δ*−^ on Pd and CuPd surfaces. The results suggest that the mechanism of CO_2_ hydrogenation towards the formate species may be surface dependent: the more exposed H on the PdZn surfaces, relative to Pd, means CO_2_ can react with H* directly from the gas phase, *i.e.*, an Eley–Rideal-type mechanism, rather than the Langmuir–Hinshelwood mechanism.

Forming HCOO from physisorbed CO_2_ on Pd surfaces has a higher *E*_a_ than its decomposition, with *E*_a_(CO_2_ + H), 0.33, 0.11 and 0.04 eV greater on Pd (111), (100) and (110), respectively. Cu surfaces show a lower *E*_a_(CO_2_ + H), and hence formate remains stable once the carbon is hydrogenated. On Zn (0001), a low *E*_a_(CO_2_ + H) of 0.54 eV is calculated assuming a chemisorbed H, but the TS on Zn (0001) is noted as being the highest in energy across all modelled surfaces, with respect to gas substrates, due to the unfavourable Zn–H interaction, visible on the reaction energy profile in Fig. 6. Consideration of the chemisorbed CO_2_^*δ*−^ species for PdZn(101) gives a low *E*_a_(CO_2_^*δ*−^ + H) of 0.30 eV, and the barrierless *E*_a_(CO_2_^*δ*−^ + H) on PdZn (110), indicates that transfer of the H to form a C–H bond requires less energy than on Pd, and the relative *E*_ads_ of CO_2_ and CO_2_^*δ*−^ constitutes most of the barrier. We can therefore conclude that the CO_2_ activation remains challenging on the surface of the pristine PdZn surfaces, but hydrogenating CO_2_ is more efficient than on Pd.

To aid analysis, Pearson correlation was calculated for geometric and energetic quantities related to reactants, products, and TS structures, as shown in Table S4 the SI. The geometries and energies of TS structures are observed to be uncorrelated with the properties of the initial structures in the reactions; however, a positive linear correlation (0.94) of *E*_a_(CO_2_ + H) and *E*_ads_ of HCOO^m^* is observed across Pd (111), (100), (110), Cu (100), (110), CuPd (110), PdZn (101) and (110) surfaces (Fig. S3 in the SI). Simple relationships between adsorbed intermediates and reaction energetics have been previously used successfully to understand and predict catalytic activity for electrochemical CO_2_ reduction.^68,69^ The trends presented are promising for investigating other Pd-based alloys, where modelling of HCOO^m^* could be used to efficiently estimate the *E*_a_(CO_2_ + H) without running expensive NEB calculations for transition state searches.

Here, the correlation can be considered meaningful as the HCOO^m^* structure captures the interaction of the surface with hydrogen and with the oxygen atoms in the intermediates. The Hammond postulate, coupled with the observed reaction energetics, reasserts that relationship of the HCOO^m^* to the TS is sensible, as endergonic reactions have TS structures more akin to the product than the reactants. The surface on which no metastable HCOO^m^* was found, Zn (0001), has shown no metastable CO_2_^*δ*−^ and overall large *E*_a_(CO_2_ + H); thus, the absence of the metastable geometry is also informative, as it confirms that the product is unlikely to form. From the trends observed, *E*_ads_(HCOO^m^)* emerges as an important target property for further SGD investigation, supporting consideration of catalytically active Pd-based alloys for CO_2_ hydrogenation to formate. The chemical context is critical for SGD-supported simulation design and meaningful interpretation of the SGD-derived rules.

### 3.4 Pd single-atom alloys

To broaden the design options for metal catalysts, the observations in Sections 3.1–3.3 were used as a platform for an SGD analysis of the HCOO^m^* adsorbed on SAA Pd surfaces. The investigation focused on Pd–M alloy candidates that facilitate the desired formate pathway of CO_2_ hydrogenation, with the correlation between the *E*_ads_(HCOO^m^*) and *E*_a_(CO_2_ + H) (Fig. S3) allowing us to use *E*_ads_(HCOO^m^*) as a target property.

A dataset was prepared by performing a survey of HCOO^m^* adsorption across a range of single-atom alloy Pd (111), (100), (110) and (211) surfaces. The results were filtered to ensure that the adsorbate does not scission and the hydrogen does not dissociate from the surface, ensuring that one H–M and at least one O–M bond exists, where M is one of the following species considered: Pd (*i.e.*, non-alloy surface), Co, Cu, Ga, Ir, Ni, Os, Pd, Pt, Rh, Ru, or Zn. The resulting dataset contains 49 symmetrically inequivalent structures, with the lowest *E*_ads_ for each SAA shown in Table S5 in the SI.

**Fig. 7:**
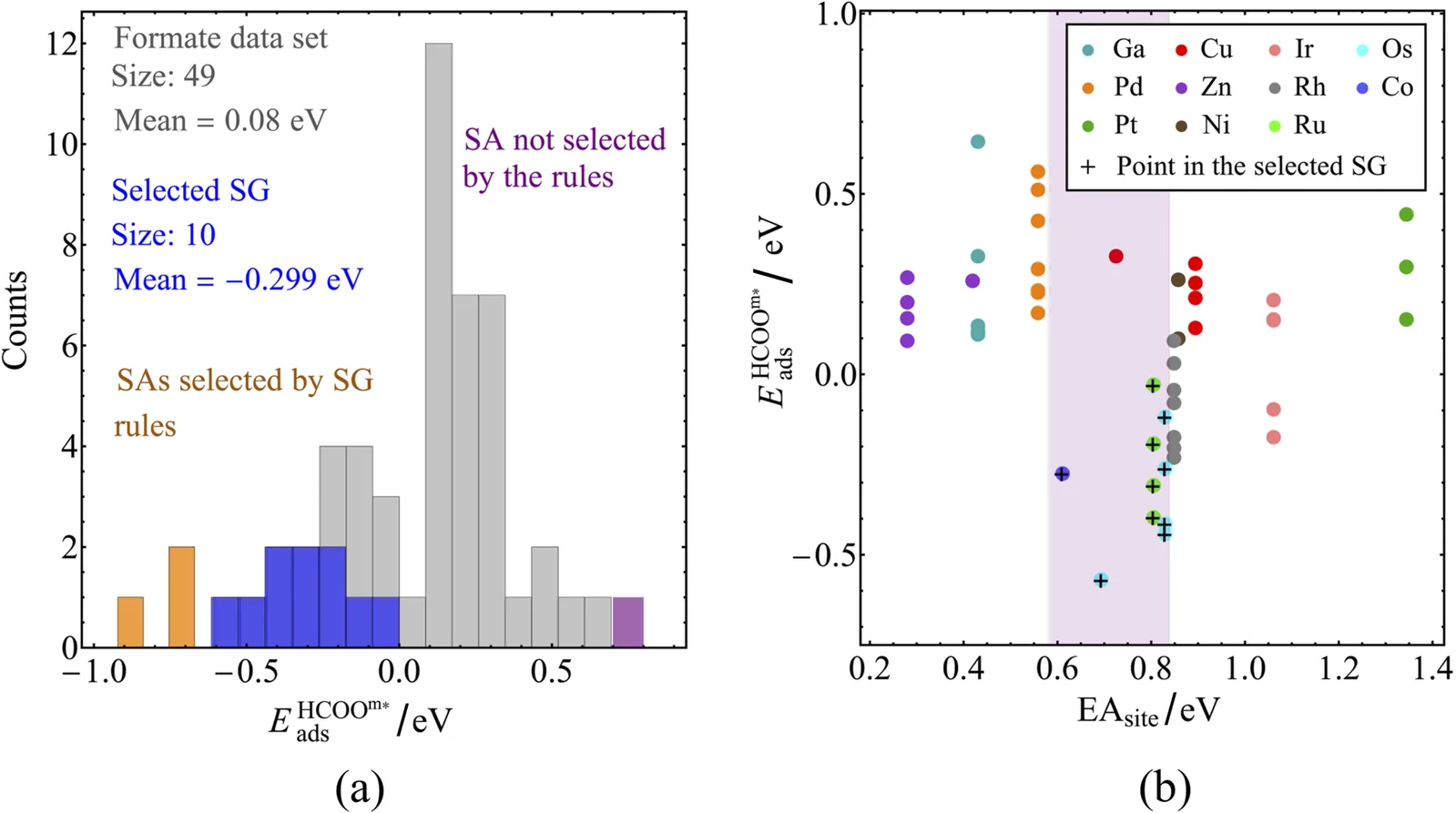
(a) Adsorption energy distribution of the HCOO^m^* data set (grey bins) and of the subgroup identified by SGD (blue bins). The bins containing SAs tested after screening for candidate SAAs are shown in orange (Nb, Mo, W) and purple (Ge). (b) Adsorption energy of HCOO^m^* on SAAs with respect to the average electron affinity of the metal atoms interacting with the adsorbate (EA_site_), one of the key parameters of the selected SG. The highlighted region defines the thresholds given by the SG rules.

From the 49 structures considered, 32 result in *E*_ads_(HCOO^m^*) > 0 eV (Fig. 7a). To identify subgroups (SGs) of the SAAs that strongly bond HCOO^m^*, the normalized negative mean shift utility function was applied when screening data, as described in Section S5 in the SI. Thus the resulting SGD search favours subgroups (SGs) with low mean target values (*i.e.*, exothermic). The SG with 10 data points and desirable average *E*_ads_(HCOO^m^*) of −0.30 eV is identified (Fig. 7a, blue bins). The SG includes bridge sites of the (111) and (100) surface, the short and long bridges in the (110) termination, and the bridges located at the step of the (211) surface. The SG includes 3 SAs that are associated with the favourable performance: Co, Ru, and Os. Os is used for homogeneous CO_2_ hydrogenation^70,71^ but the performance of supported Os heterogeneous nanoparticle catalysts for CO and CO_2_ reduction is not strong.^72,73^ Ru is a well-known hydrogenation catalyst, and supported Ru nanoparticle catalysts have excelled for CO_2_ hydrogenation to formate.^74^ A significant downside of Os and Ru is they are inherently scarce, similar to Pd, and therefore identifying more earth-abundant metals catalysts for CO_2_ hydrogenation with the subgroup design rules is required.

The SGD identifies a conjunction (“∧”) of two inequalities that define the selected SG:EA_SA_ ≤ 1.192 eV ∧ 0.583 ≤ EA_site_ ≤ 0.838 eV,where EA is the electron affinity and the subscript denotes the property belongs to the dopant (SA) or the surface (site). From a chemical perspective EA_SA_ and EA_site_ are sensible as they measure the active site’s tendency to withdraw electrons, impacting the charge distribution and its redox flexibility providing means of intermediate stabilisation. Applying the SGD rules allows the search of SAAs suitable for providing strong surface-HCOO^m^* binding, which is our focus as it facilitates the Eley–Rideal mechanism and removes the pre-requisite of CO_2_ chemisorption for the formate CO_2_ hydrogenation pathway.

To further our investigation, the bridge site of Pd (100) was combined with 22 new SA elements for candidate screening. The dopants considered are Sc, Ti, V, Cr, Mn, Fe, Ge, Y, Zr, Nb, Mo, Ag, Cd, In, Sn, Hf, Ta, W, Re, Au, Hg, and Tl. Applying the SG rules reduces the viable SAAs to 4 dopants in this new sample: Cr, Nb, Mo, and W. The validity of the predictions are confirmed by DFT calculations (Table 2), and the *E*_ads_(HCOO^m^*) for the SAA with Nb, Mo, and W is very favourable at −0.86, −0.75, and −0.75 eV, respectively (Fig. 7a orange bins). The calculated adsorption energies are below the minimum value in the training data set. To prevent confirmation bias, we also tested two poorly performing dopants not selected by the SGD rules, Ge and Ag. DFT calculations resulted in unfavourable SAA candidates, with the Ge SAA resulting in a highly endothermic metastable structure, with *E*_ads_(HCOO^m^*) of 0.70 eV (Fig. 7a purple bin), and no metastable HCOO^m^* was observed on the Ag SAA. The DFT calculations confirm the value of the SG rules for identifying dopants capable of enhancing the interaction of HCOO^m^* with Pd-based SAAs.

**Table 2:** Results for the screening of dopants in SAAs with strong binding to HCOO^m^*. The rules identified by SGD correctly predict strong interactions for the selected SAAs with Nb, Mo, and W. Moreover, those SAs not selected by the rules, Ge and Ag, do not provide stable HCOO^m^* structures. Dashes denote where no minima HCOO^m^* structures found after optimisation

Surface	Adsorption site	SA	Selected by SG rules?	*E*_ads_(HCOO^m^*)/eV
Pd (100)	Bridge	Nb	Yes	−0.86
		Mo		−0.75
		W		−0.75

		Ge	No	0.70
		Ag		—

Though Pd-based alloy catalysts with Mo, No and W are not clearly visible in the literature, several studies have explored Pd catalysts on Mo-, Nb- and W-based materials for CO_2_ hydrogenation in various types of catalytic CO_2_ hydrogenation process. In terms of thermocatalytic CO_2_ hydrogenation, Mo_2_C-supported Pd nanoparticles show significantly enhanced activity, with lower apparent activation energies for CO_2_ hydrogenation to formate than their Ru–Pd/Mo_2_C counterparts (where Ru merely stabilises the Pd NPs).^75^ Likewise, Pd/MoO_3_ catalysts show substantially higher catalytic activity than Cu/ZnO catalysts in liquid-phase CO_2_ hydrogenation under mild conditions.^76^ Pd/NbC and Pd/WC materials have been reported as potent CO_2_ electroreduction catalysts, owing to favourable Pd–M electronic interactions.^77^ In thermo-photo-catalytic reverse water–gas shift reaction (RWGS), small Pd NPs on Nb_2_O_5_ are highly selective towards CO, suppressing CH_4_ formation typical of Pd catalysts.^78^ Finally, Nb-doped Pd/TiO_2_ also demonstrates synergistic thermo-photo-catalytic production of syngas (CO + H_2_) from methanol/water mixtures, with notable effects even at 0.5 wt% loadings.^79^

In summary, there are examples where the Mo, Nb and W atoms may play an important role for CO_2_ hydrogenation; Mo–Pd, Nb–Pd, W–Pd alloy nanoparticle-based catalysts for CO_2_ reduction are not well explored, and the metal oxide- or metal carbide-supported Pd catalysts reported thus far are not typically thoroughly tested for the presence of these alloys. Only the PdMo alloy catalysts have been reported, with ability to hydrogenate CO_2_ to methanol at 25 °C and 9 bar pressure, which are much milder conditions than the conventional CZA catalyst, though at lower turnover rates.^80^ Computational works show that the MoNb-doped Pd surfaces with dual single-atom sites are predicted to be especially active for CO_2_ activation^42^ and near-surface Pd/W alloys favour the formation of HCOO* over COOH* in electrocatalytic CO_2_ reduction.^81^

In summary, the subgroup discovery has been successfully applied to identify promising Pd–M alloy candidates for CO_2_ hydrogenation to methanol. Mo, Nb and W are highlighted as potent alloy candidates enhancing the selectivity of Pd catalysts by facilitating the Eley–Rideal mechanism of CO_2_ hydrogenation to formate. The mechanistic change alleviates the challenge of CO_2_ pre-activation and allows the reaction to proceed *via* the formate pathway of methanol synthesis. The Pd–M alloy SGD analysis presented herein provides strong evidence of enhanced CO_2_ activation towards formate but is not informed on further mechanistic aspects of the reaction, which could ultimately dictate the selectivity of CO_2_ hydrogenation on these alloy catalysts. The present calculations neglect several catalytically relevant factors, including the influence of temperature and pressure on surface composition and reaction pathways; future work should therefore explicitly account for these effects. Future first-principles studies are required to confirm the viability of Mo–Pd, Nb–Pd, Pd–W alloy formation and stability under reaction conditions, and to explore further the mechanistic aspects of CO_2_ hydrogenation on these materials.

## 4 Conclusions

The synthesis of methanol from CO_2_ and hydrogen using Pd-based alloy catalysts holds great potential for sustainable energy storage. Gaining deeper knowledge of the initial stages of CO_2_ hydrogenation on Pd-based catalysts is essential for understanding the variation in reactivity observed upon alloying, which is important for the strategic design of novel catalysts. Here, we have investigated the initial CO_2_ hydrogenation to formate across monometallic FCC Pd (111), (100), (110), FCC Cu (111), (100), (110), HCP Zn (0001) surfaces and BCC CuPd (110) and BCT PdZn (101) and (110) alloy facets. Cu and PdZn surfaces have the lowest hydrogenation barriers. CuPd (110) has a similar activation energy to Pd surfaces, but the formate product is much less stable on the surface. CO_2_ activation constitutes a major part of the CO_2_ hydrogenation barrier on PdZn and Cu surfaces, thus making the chemical pre-adsorption of CO_2_ unlikely and suggests an Eley–Rideal mechanism as opposed to the Langmuir–Hinshelwood model. Statistical analysis shows that there are limited correlations between reactants and transition state structures, but the adsorption energy of the HCOO^m^ product does correlate with the activation energy required for hydrogenation of the CO_2_ species.

Subgroup discovery was applied on a dataset derived from a HCOO^m^* adsorption survey on a range of SAA Pd (111), (100), (110) and (211) surfaces, doped with Co, Cu, Ga, Ir, Ni, Os, Pd, Pt, Rh, Ru or Zn. The chosen 13 candidate features relating to the dopant or the adsorption site of HCOO^m^* were used to capture the SAA surfaces’ electronic and geometric properties. The combination of DFT and SGD analysis shows that the electron affinity (EA) of the dopant, which is an intrinsic material property, can be used to predict which SAA surfaces facilitate HCOO^m^* adsorption. The resulting selectivity criteria allows us to identify Nb, Mo, and W as candidates to alloy with Pd catalysts and significantly improve the capability of CO_2_ hydrogenation to formate. The materials have the potential to enable the formate pathway of methanol synthesis on Pd-based catalysts with much improved precious metal utilisation.

The workflow and results from this investigation can lead the way for theory-led rational design of alloy catalysts, in this case specifically targeting mechanistic aspects of CO_2_ hydrogenation. The general workflow offers potential applicability to other reactions making use of alloy catalysts, accelerating catalyst discovery.

## Author contributions

Igor Kowalec: conceptualisation, data curation, formal analysis, investigation, methodology, resources, validation, visualisation, roles/writing – original draft; Herzain Rivera Arrieta: data curation, formal analysis, methodology, resources, software, roles/writing – original draft; Zhongwei Lu: data curation, formal analysis, roles/writing – original draft; Lucas Foppa: formal analysis, methodology, software, supervision, writing – review & editing; Matthias Scheffler: formal analysis, funding acquisition, methodology, software, supervision, writing – review & editing; Richard Catlow: conceptualisation, funding acquisition, methodology, supervision, writing – review & editing; Andrew Logsdail: conceptualisation, formal analysis, funding acquisition, methodology, project administration, resources, supervision, writing – review & editing.

## Conflicts of interest

The authors declare that they have no known competing financial interests or personal relationships that could have appeared to influence the work reported in this paper.

## Supplementary Material

FD-267-D5FD00125K-s001

## Data Availability

The inputs and output files of all electronic structure calculations have been uploaded as a dataset to the NOMAD repository at DOI: https://doi.org/10.17172/NOMAD/2026.02.02-1. Supplementary information (SI) is available. See DOI: https://doi.org/10.1039/d5fd00125k.
