# Supplementary material for: Role of monodentate formate in product selectivity for CO2 hydrogenation on Pd-based alloy catalysts
Source: Faraday Discuss. 2026 Feb 3. Online ahead of print. doi: 10.1039/d5fd00125k (PMC13159975; doi:10.1039/d5fd00125k)
Supplement: FD-OLF-D5FD00125K-s001 [file FD-OLF-D5FD00125K-s001.pdf]

Supplementary Information (SI) for Faraday Discussions.
This journal is © The Royal Society of Chemistry 2026

Supporting Information:

Role of monodentate formate in product selectivity for CO_2 hydrogenation on Pd-based alloy catalysts

Igor Kowalec[1,*], Herzain I. Rivera-Arrieta[2], Zhongwei Lu[1], Lucas Foppa[2], Matthias Scheffler[2], C. Richard A. Catlow[1,3,4], Andrew J. Logsdail[1,*]

[1] Cardiff Catalysis Institute, School of Chemistry, Cardiff University, Park Place, Cardiff, CF10 3AT, Wales, United Kingdom

[2] The NOMAD Laboratory at the Fritz Haber Institut of the Max Planck Society, Faradayweg 4-6, Berlin 14195, Germany

[3] Kathleen Lonsdale Materials Chemistry, Department of Chemistry, University College London, London WC1H 0AJ, United Kingdom

[4] UK Catalysis Hub, Research Complex at Harwell, Rutherford Appleton Laboratory, Harwell, Oxon OX11 0FA, United Kingdom

Emails: KowalecI@cardiff.ac.uk; LogsdailA@cardiff.ac.uk

S1. Metallic, alloy and SAA surfaces - geometry optimisation details

The monometallic and alloy surfaces used in Sections 3.1 – 3.3 are presented in S1.

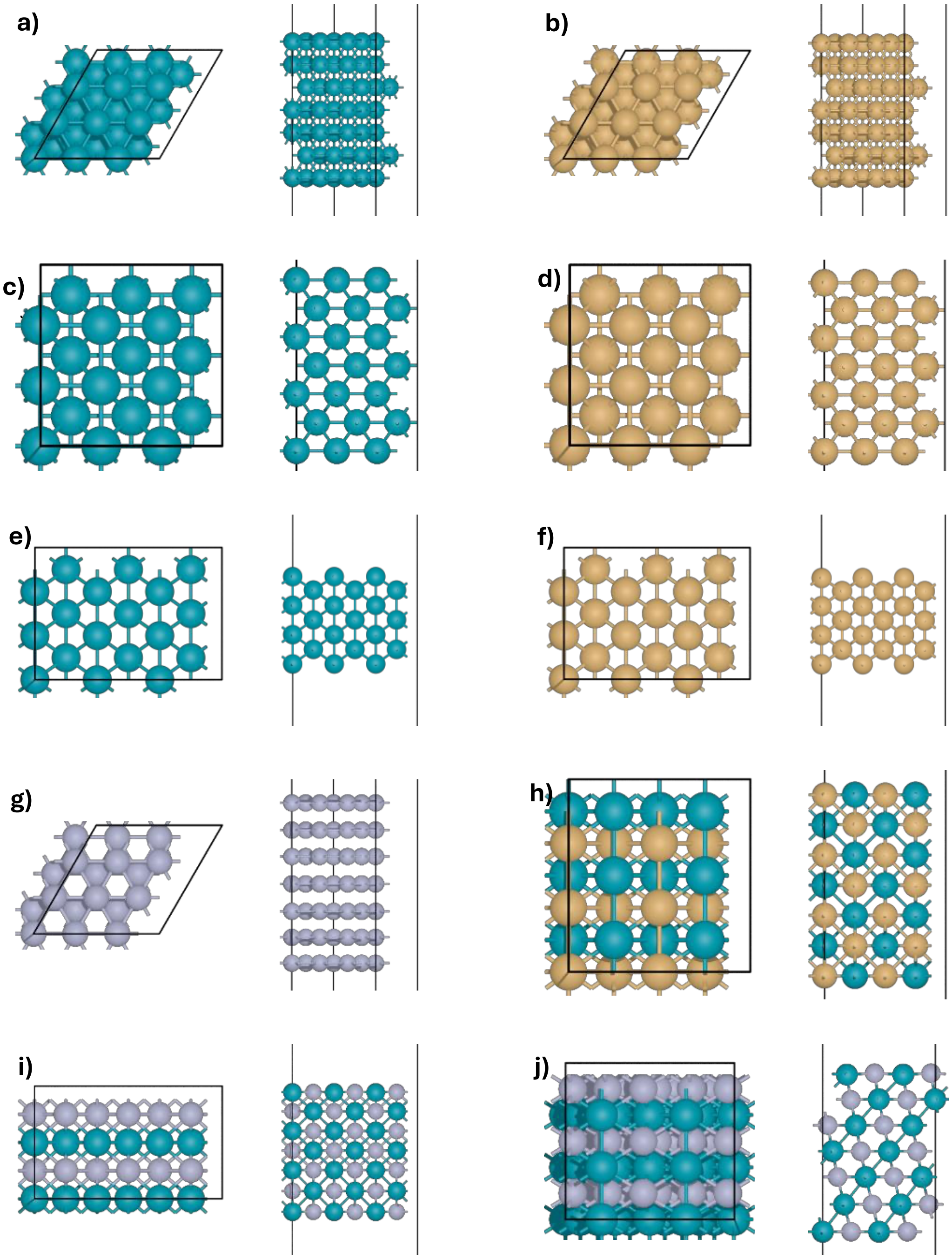

Figure S1. The pristine metallic and alloy surfaces used in the study, with top- and side-view presented on the left- and right-hand side, respectively: a) FCC Pd (111), b) FCC Cu Pd (111), c) FCC Pd (100), d) FCC Cu (100), e) FCC Pd (110), f) FCC Pd (110), g) HCP Zn (0001), h) BCC CuPd (110), i) BCT PdZn (101), j) BCT PdZn (110).

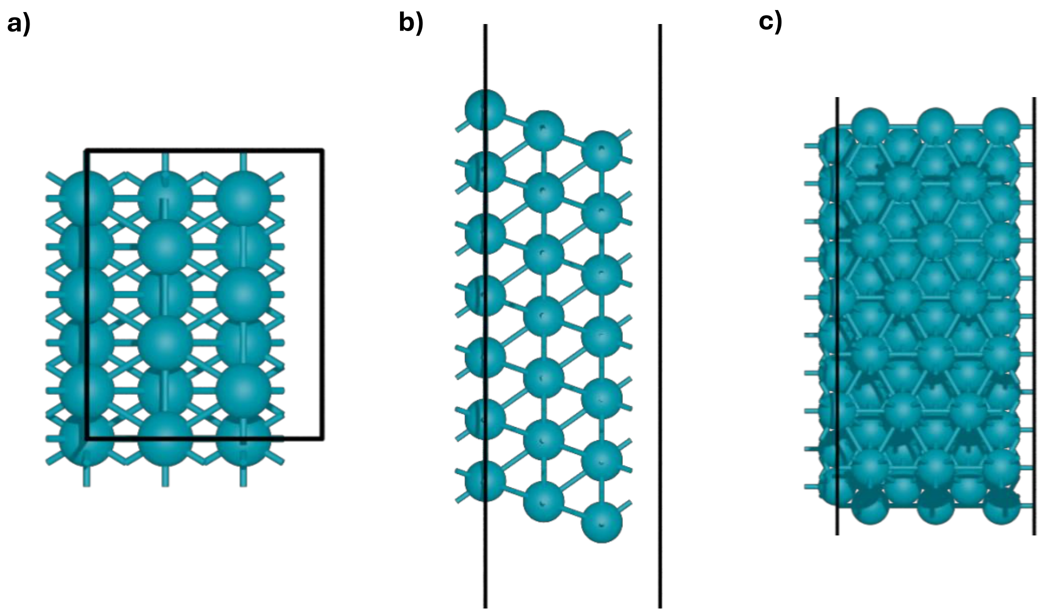

Figure S2. The pristine monometallic FCC Pd (211) surface used in the single-atom alloy study viewed in the *xy*-, *xz*- and *yz*-plane in a, b and c, respectively.

S1.2. Construction of system models for geometry optimisation

Starting $HCOO^{m*}$ geometries were obtained by adjusting the chemisorbed CO_2 adsorbate height, introducing the hydrogen atom and scaling molecular bond lengths and bond angles based on scaling factors from the reference chemisorbed CO_2 structures and monodentate formate on Pd (111) surface. To accelerate the calculation workflow prior to DFT, the monodentate formate geometries on Pd SAA surfaces were initially optimised with the MACE forcefield[1] with the "small" machine-learned forcefield and a van der Waals forces correction.[2,3] A convergence criterion of maximum forces below 0.05 eV $Å^{-1}$.

The structures were optimised in a two-step manner: firstly, the surface was fully constrained along with the oxygen nearest to the surface to anchor the adsorbate; and in the second pre-optimisation step, the constraint on the oxygen and nearest metal neighbours to the adsorbate was lifted. The resulting MACE-optimised structures were subsequently filtered to remove bidentate formate and formate dissociated to CO_2 and H, and symmetrical equivalent structures. The MACE-optimised monodentate formate structures on Pd SAAs were then optimised using DFT, with settings and constraints identical to those of the clean surfaces.

S2. Mulliken charge analysis of adsorbates on Cu, Pd and CuPd (110) surfaces

Mulliken charge analysis was performed for the discussed intermediates on FCC Cu (110), FCC Pd (110), BCC CuPd (110), BCT PdZn (101) and BCT PdZn (110) surfaces to rationalise trends in E_{ads}. The changes in atomic charge (Δq) are reported in electrons (e) with respect to the atomic charges of each atom on the pristine surfaces; the sum of Δq over the whole slab (Δq_{slab}), average of Δq per atom in the first surface layer (Δq_{surf}), maximum Δq of a single atom in the surface layer (Δq_{surf_max}) and the charges on the C (q_C) and H (q_H, where available) are tabulated in Table S1. Positive values of Δq indicate charge transfer from the metal to the molecule. Table S1 also contains the oxygen-carbon-oxygen angles (∠OCO) of the adsorbates to allow comparison of structural features with electronic.

Table S1 shows that both Δq_{slab} and Δq_{surf} were negligible for CO_2 physisorbed on the surfaces, which is because there is no charge transfer in physisorbed systems; there is also limited change of (< 0.3 °) in the ∠OCO from the 180 ° configuration of gas phase CO_2. The Δq_{slab} of 0.31 e on the Pd (110) facet for $CO_2^{\delta-}$ adsorption aligns with the 0.28 e calculated for the Pd (111) surface by Tang *et al.*,[4] and is greater than our own previous result of 0.11 e for the Pd (110) surface.[5] The difference for results on the Pd (110) surface is attributed to differences in the applied exchange correlation functional, and changes to the depth of the surface slab model and number of relaxed atomic layers, thus affecting the perceived ability of the Pd bulk layers in donating electrons towards the surface layers.[5,6] The number of layers was found to be critical in accurate representation of the Cu surfaces also.[6]

The $HCOO^m$ intermediate accepts a significant amount of charge from the surface slabs and maintains an almost identical q_C as the $HCOO^b$ structures on corresponding surfaces. The q_H in $HCOO^m$ structures is consistently lower (more electrons) than in $HCOO^b$ across investigated facets, signifying a meaningful electronic interaction between the surfaces and the hydrogen atom in the $HCOO^m$ species.

Table S1. Change in Mulliken charges, in units of e, for atoms in the surface slab when CO_2, $CO_2^{\delta-}$, $HCOO^b$ and $HCOO^m$ are adsorbed on Cu (110), Pd (110), CuPd (110), PdZn (101) and (110) surfaces, relative to the atomic charges on the pristine surface. The charge differences (Δq) have been averaged over the first surface layer (Δq_{surf}), summed over the whole slab (Δq_{slab}), and quantified with respect to the maximum change in the atomic surface atomic layer charge (Δq_{surf_max}). The OCO angle (∠OCO) in the adsorbate is also shown in units of degrees (°). Provided are also final charge on the carbon (q_C) and the hydrogen atom (q_H), where available.

Surface	Structure	Δq_{slab} /e	Δq_{surf} /e	Δq_{surf_max} /e	∠OCO / °	q_C /e	q_H /e
**Cu (110)**	$CO_2^{\delta-}$	0.01	0.01	0.03	179.8	0.54	-
	CO_2	0.48	0.06	0.19	127.3	0.28	-
**Pd (110)**	$CO_2^{\delta-}$	0.01	0.0	0.02	179.7	0.54	-
	CO_2	0.31	0.05	0.21	137.4	0.41	-
**CuPd (110)**	$CO_2^{\delta-}$	0.01	0.0	0.04	179.8	0.54	-
	CO_2	0.26	0.03	0.17	140.4	0.40	-
**PdZn (101)**	$CO_2^{\delta-}$	0.01	0.00	0.03	179.8	0.55	-
	CO_2	0.38	0.04	0.23	135.3	0.36	-
**PdZn (110)**	$CO_2^{\delta-}$	0.01	0.00	0.02	179.9	0.55	-
	CO_2	0.36	0.04	0.26	137.7	0.37	-
**Cu (110)**	$HCOO^m$	0.46	0.07	0.20	129.8	0.34	0.00
	$HCOO^b$	0.47	0.06	0.20	127.2	0.33	0.08
**Pd (110)**	$HCOO^m$	0.35	0.06	0.23	132.3	0.35	0.04
	$HCOO^b$	0.43	0.06	0.21	128.8	0.34	0.09
**CuPd (110)**	$HCOO^m$	0.37	0.04	0.18	132.2	0.33	0.05
	$HCOO^b$	0.43	0.04	0.17	128.5	0.32	0.09
**PdZn (101)**	$HCOO^m$	0.50	0.04	0.25	128.9	0.33	0.02
	$HCOO^b$	0.53	0.05	0.24	128.0	0.33	0.08
**PdZn (110)**	$HCOO^m$	0.50	0.05	0.29	128.7	0.32	0.04
	$HCOO^b$	0.54	0.05	0.28	127.7	0.33	0.08

S3. Transition states discussion

The impact of the geometry of carbon dioxide and formate on the subsequent transition states was assessed here by performing a transition state (TS) search on the PdZn (110) surface considering the CO_2 or $CO_2^{\delta-}$ adsorbed structures, and co-adsorbed hydrogen atom, as the initial geometry to form either $HCOO^{b*}$ or $HCOO^{m*}$. The results are presented in Table S2.

Table S2. Comparison of how changes in the geometry of CO_2 reactant influences the resulting transition state (TS) energy and geometry when proceeding to the monodentate and bidentate formate product. The activation energy (E_{act}, eV), the length of the *M*-C and C-H bonds at the transition state (d_{TS}, Å), and the angle of the O-C-O adsorbate bonds ($\angle_{TS}$, °) are given. The length of the minimum energy path (d_{MEP}, Å) is also given. A hyphen denotes lack of data due to a non-convergent calculation.

Reactants	Products	d_{MEP}	E_{act}	$d_{TS}(Pd–C)$	d_{TS}(C–H)	$\angle_{TS}$
$*CO_2 + *H$	$HCOO^{m*}$	5.4	0.49	3.30	1.68	153.1
$*CO_2^{\delta-} + *H$	$HCOO^{m*}$	7.1	0.50	2.95	1.71	152.2
$*CO_2 + *H$	$HCOO^{b*}$	11.5	0.54	3.30	1.64	151.1
$*CO_2^{\delta-} + *H$	$HCOO^{b*}$	-	-	-	-	-

For reactions that start from physisorbed CO_2, the Pd-C distance in the TS is 0.35 Å longer than when starting with the chemisorbed $CO_2^{\delta-}$, but there are only small differences in the length of the C-H bond (< 0.07 Å). Similarly, the activation energy differs by 0.05 eV or less for the different reaction pathways. The $CO_2^{\delta-}$ and H* to $HCOO^{b*}$ product simulation was not performed as the atomic coordinate shift is tripled with respect to the shortest path presented, with prohibitive cost when sampled at target sampling density of 0.25 Å^{-1}. For the successful formation of $HCOO^{b*}$ from physisorbed CO_2, a monodentate $HCOO^{m*}$ species is formed as an intermediate, similar to the mechanism on Cu (110) proposed by Wang *et al.*[7] Based on the results in Table , the shorter CO_2 to $HCOO^{m*}$ reaction process can be investigated to study reaction kinetics.

S4. Pearson Correlation

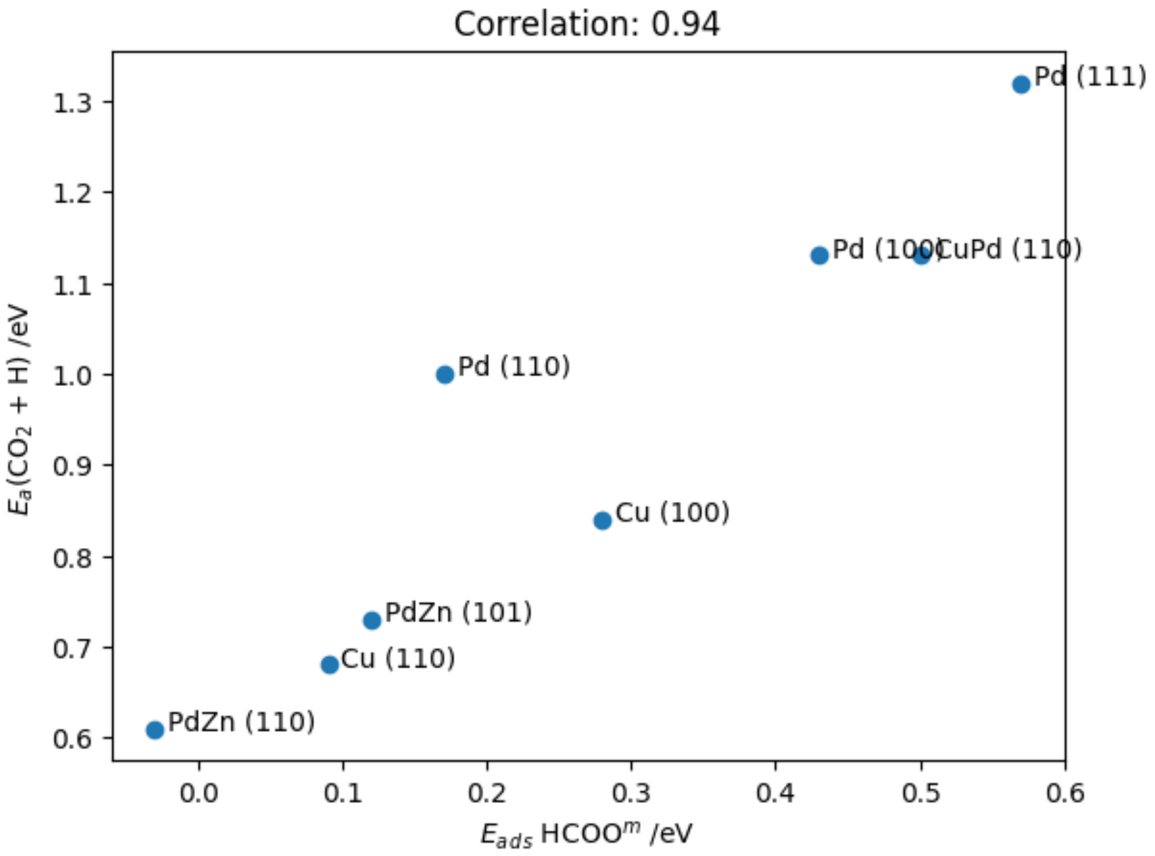

Figure S3. Pearson correlation of $E_a(CO_2 + H)$ and E_{ads} of $HCOO^m$ across Pd (111), (100), (110), Cu (100), (110), CuPd (110), PdZn (101) and (110) surfaces. The relationship has a positive Pearson correlation of 0.94 and the linear coefficient of determination, R^2, is 0.88.

S5. SGD details

Subgroup Discovery (SGD) is a focused Artificial Intelligence approach devoted to finding subsets or subgroups displaying exceptional patterns within a data set of population *P*.[8] In this work, we used the realKD software to perform our SGD studies.[9] Among the available algorithms in the program, we use the Monte Carlo search algorithm. Two parameters need to be chosen for a SGD run: the number of k-means used to section the candidate descriptive parameters space; and the number of random seeds used to perform the stochastic search of the SGs. We used $k = 12$ as this has proven to be a reliable choice from previous studies on the CO_2 activation on SAAs.[10] The number of seeds was set to 50,000.

Rating the exceptionality of the studied SGs is based on the maximization of a quality function $Q(S, P)$:

$$Q(S, P) = s(S)s(P) \cdot u(S, P),$$

where $s(S)s(P)$ is the ratio between the size (s) of the subgroup (S) and the entire population (P). This term, known as the coverage, helps to avoid the selection of SGs containing too few points. The second term, $u(S, P)$, is the utility function and allows addressing the target of interest Y. As we aim to identify SGs of SAAs displaying strong binding of the $HCOO^{m*}$, we used the *normalized negative mean shift* as the utility function.

$$u(S, P) = Y(P)\text{-}Y(S)Y(P)\text{-}Y_{min}(P) \ .$$

Instead of a direct selection of the SG with the maximum $Q(S, P)$ value, we performed a Pareto front analysis of the top-rated solutions provided by SGD.[11] This approach considers different trade-offs among the set of optimal solutions for the two conflicting objectives coverage and utility. As the coverage of the SGs in the Pareto front increases, the rules will provide more general descriptions. On the other hand, lower coverages allow the scrutiny of the more exceptional situations. A comparison of the SGs in the Pareto front is provided in Table S3. From the pool of offered candidate descriptive parameters, the electron affinities from the SA and the adsorption site (EA_{SA}, EA_{site}) are key properties holding a connection with $E_{ads}(HCOO^{m*})$. Not surprisingly, as the coverage increases, the thresholds and constraints are

looser and more SAs belong to such SGs. In addition to the EA, the SA s-orbital and valence radii are relevant parameters to define different SGs in the Pareto front.

Figure S2 shows the SGs considered in this study and their coverage (*x*-axis) and utility (*y*-axis) values. The magenta points represent the Pareto front SGs. The green point is the SG maximizing the quality function and has 22 data points, with an average $E_{ads}(HCOO^{m}*)$ of 0.08 eV. We aimed for the more exceptional situations, *i.e.*, SGs with high utility values; however, the SG with the highest utility only contains Ru and Os, and using the rules that define this SG might be too restrictive when searching for candidate SAAs for the $E_{ads}(HCOO^{m}*)$ stabilization. We therefore selected the second point in the Pareto front as the representative SG in this study, as it provides a slightly wider SAs chemical space (Co, Ru, Os). With 10 data points and a mean target of –0.299 eV, the blue point in Figure S4 corresponds to the selected SG.

Table S3. Coverage, utility, rules, and SAs in the Pareto front of SGs. The surface terminations and adsorption sites in these subgroups are Pd(111)-bridge, Pd(100)-bridge, Pd(110) short and long bridges, and Pd(211)-bridges (step).

Pareto front index	Coverage	Utility	SG rules	SAs in the SG
1	0.184	0.589	EA_{sa} eV ≤ 1.192 ⋀ $r_{val\text{-}SA}$ ≥ 0.63 Å ⋀ $r_{s\text{-}SA}$ ≥ 1.261 Å	Ru, Os
2 (Sel. SG)	0.204	0.584	0.583 ≤ EA_{site} ≤ 0.838 eV ⋀ EA_{SA} ≤ 1.192 eV	Co, Ru, Os
3	0.265	0.510	0.609 ≤ EA_{SA} ≤ 1.146 eV ⋀ gen-CN ≤ 7.11	Ru, Rh, Os
4	0.286	0.454	EA_{SA} ≤ 1.146 eV ⋀ PE_{site} ≥ 2.02 ⋀ CN ≤ 6.0 ⋀ $r_{val\text{-}SA}$ ≥ 0.613 Å	Zn, Ru, Rh, Os
5	0.347	0.449	EA_{SA} ≤ 1.40 eV ⋀ EA_{site} ≥ 0.583 eV ⋀ $r_{s\text{-}SA}$ ≥ 1.261 Å	Co, Ru, Rh, Os
6	0.367	0.402	0.583 ≤ EA_{site} ≤ 0.852 eV	Co, Cu, Ru, Rh, Os
7	0.388	0.385	EA_{SA} ≥ 0.61 eV ⋀ EA_{site} ≤ 0.977 eV ⋀ $r_{val\text{-}SA}$ ≤ 1.1 Å	Co, Ni, Ru, Rh, Os
8 (Max(Q))	0.449	0.358	0.583 eV ≥ EA_{site} ⋀ EA_{SA} ≤ 1.846 eV ⋀ $r_{s\text{-}SA}$ ≥ 1.261 Å	Co, Ru, Rh, Ir, Os
9	0.490	0.315	EA_{SA} ≤ 1.565 eV ⋀ EA_{site} ≥ 0.58 eV ⋀ $r_{s\text{-}SA}$ ≥ 1.226 Å	Co, Ni, Ru, Rh, Ir, Os
10	0.510	0.274	EA_{site} ≥ 0.583 eV ⋀ $r_{s\text{-}SA}$ ≥ 1.261 Å	Co, Ru, Rh, Ir, Os, Pt
11	0.531	0.247	EA_{site} ≤ 1.565 eV ⋀ 1.1 ≤ $r_{val\text{-}SA}$ ≤ 0.597 Å	Zn, Ru, Rh, Ir, Os
12	0.551	0.242	EA_{site} ≥ 0.583 eV ⋀ $r_{s\text{-}SA}$ ≥ 1.226 Å	Co, Ni, Ru, Rh, Ir, Os, Pt
13	0.591	0.216	0.583 ≤ EA_{site} ≤ 1.202 eV	Co, Ni, Cu, Ru, Rh, Ir, Os
14	0.633	0.166	EA_{sa} ≤ 1.846 eV ⋀ 0.597 ≤ $r_{val\text{-}SA}$ ≤1.199 Å	Cu, Zn, Ru, Rh, Ir, Os
15	0.653	0.164	0.583 eV ≤ EA_{site}	Co, Ni, Cu, Ru, Rh, Ir, Os, Pt

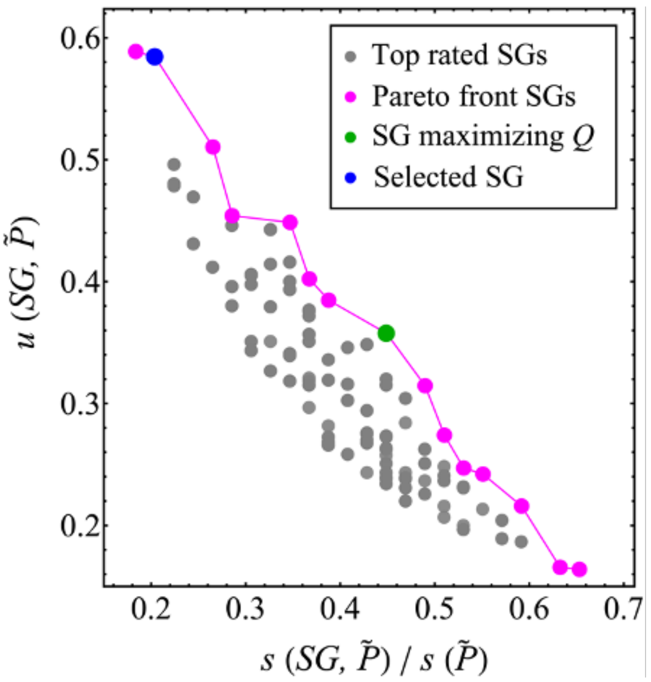

Fig. S4. Utility-coverage plot for the top-rated solutions found through SGD. The magenta points represent the SGs in the Pareto front. The green and blue points correspond to the SGs maximizing the quality function and the one selected for our analysis, respectively.

Table S4. Numerical data for CO_2 dissociation and CO_2 hydrogenation to formate on FCC Pd (111), (100), (110), FCC Cu (111), (100), (110), HCP Zn (0001), CuPd (110) and PdZn (101) and (110) surfaces; physisorbed and chemisorbed carbon dioxide species are denoted as CO_2 and $CO_2^{\delta-}$, respectively; monodentate and bidentate forms of formate intermediate are represented as $HCOO^m$ and $HCOO^b$, respectively; + denotes co-adsorption of species; E_{ads} is measured with respect to gas phase substrates (CO_2 and/or ½H_2) and pristine surfaces in units of eV; atomic bond lengths, *d*(atom–atom) are measured in Å, where M represents the metal atom closest to the second atom; ∠species(O–C–O) is the oxygen-carbon-oxygen bond angle in units of °; E_{ads} of H is reported from structures with co-adsorbed physisorbed CO_2 and chemisorbed H atom; E_a(species) is the activation energy required for the formation of a transition state, starting from the species in brackets.

Species or process	surface facet	Pd (111)	Pd (100)	Pd (110)	Zn (0001)	PdZn (101)	PdZn (110)	CuPd (110)	Cu (110)	Cu (100)	Cu (111)
$HCOO^m$	E_{ads}($HCOO^m$) /eV	0.57	0.43	0.17		0.12	-0.03	0.50	0.09	0.28	0.45
	*d*HCOO(H–M) /Å	1.784	1.771	1.788		2.310	1.987	1.882	1.846	1.878	1.918
	*d*HCOO(H–C) /Å	1.216	1.213	1.206		1.158	1.156	1.180	1.169	1.167	1.163
	*d*HCOO(M–C) /Å	2.853	2.835	2.836		3.103	2.970	2.795	2.702	2.778	2.784
	$d_1$$HCOO^m$(C–O) /Å	1.191	1.192	1.194		1.197	1.197	1.194	1.196	1.194	1.193
	$d_2$$HCOO^m$(C–O) /Å	1.263	1.261	1.263		1.284	1.285	1.277	1.276	1.279	1.284
	∠HCOO(O–C–O) /°	132.9	132.6	132.3		128.9	128.7	132.2	129.8	129.7	129.3
TS in CO_2 hydrogenation to formate	E_{ads}(TS) /eV	0.65	0.53	0.33	1.11	0.34	0.49	0.5	0.52	0.64	0.64
	*d*TS(H–M) /Å	2.00	1.78	2.06	1.68	1.68	1.68	1.67	1.57	1.58	1.501
	*d*TS(H–C) /Å	1.16	1.23	1.15	1.63	1.60	1.68	1.43	1.70	1.63	1.71
	*d*TS(M–C) /Å	2.65	2.39	2.49	3.05	2.99	3.31	2.97	2.67	2.86	2.77
	d_1TS(C–O) /Å	1.21	1.21	1.21	1.17	1.17	1.17	1.18	1.17	1.17	1.176
	d_2TS(C–O) /Å	1.28	1.27	1.28	1.2	1.2	1.2	1.22	1.18	1.2	1.216
	∠TS(O–C–O) /°	128.5	133.1	127.2	152.3	149.7	153.1	145.5	150.9	151.2	146.4

**Species or process**	**surface facet**	**Pd (111)**	**Pd (100)**	**Pd (110)**	**Zn (0001)**	**PdZn (101)**	**PdZn (110)**	**CuPd (110)**	**Cu (110)**	**Cu (100)**	**Cu (111)**
**H (from co-adsorption of CO_2 and H)**	E_{ads}**(H) /eV**	-0.53	-0.46	-0.50	0.67	-0.23	0.03	-0.47	-0.01	-0.06	-0.21
**CO_2 + H**	E_{ads}**($CO_2^{\delta-}$ + H) /eV**	-0.32	-0.50	-0.49		0.04	0.49	-0.23	0.34	0.43	
	E_{ads}**(CO_2 + H) /eV**	-0.67	-0.60	-0.67	0.57	-0.39	-0.12	-0.63	-0.18	-0.2	-0.34
**CO_2**	E_{ads}**(CO_2) /eV**	-0.14	-0.14	-0.17	-0.10	-0.16	-0.15	-0.16	-0.17	-0.14	-0.14
	E_{ads}**($CO_2^{\delta-}$) /eV**	0.15	-0.08	-0.25		0.22	0.18	0.09	0.30	0.39	
	d**$CO_2^{\delta-}$(M–C) /Å**	2.04	2.01	2.00		2.13	2.06	2.14	2.00	2.06	
	d_1**$CO_2^{\delta-}$(C–O) /Å**	1.19	1.2	1.21		1.19	1.2	1.19	1.24	1.2	
	d_2**$CO_2^{\delta-}$(C–O) /Å**	1.24	1.24	1.23		1.25	1.24	1.22	1.25	1.31	
	**∠$CO_2^{\delta-}$ (O–C–O) /°**	139.1	139.1	137.3		137.3	137.5	144.8	128.3	127.4	
**CO_2 hydrogenation**	E_a**(CO_2 + H) /eV**	1.32	1.13	1.00	0.54	0.73	0.61	1.13	0.68	0.84	0.98
	E_a**($CO_2^{\delta-}$ + H) /eV**	0.97	1.03	0.99		0.30	0.00	0.73	0.17	0.21	
	E_a**($HCOO^b$ dissociation) /eV**	0.99	1.02	0.96	1.74	1.15	1.36	1.00	1.59	1.51	1.22

Table S5. Lowest E_{ads} (eV) of monodentate formate found on the single-atom doped Pd (100), (110), (111) and (211) facets corresponding to the identity of the dopant species. *M* corresponds to the dopant element in each case. – denotes systems where no $HCOO^{m*}$ structure meeting the selection criteria was found.

M	(100)	(110)	(111)	(211)
Co	-	-1.38	-0.27	-
Cu	0.31	0.21	-	0.13
Ga	0.33	0.12	0.65	0.11
Ir	0.15	-0.17	0.21	-0.10
Ni	-	0.26	0.10	-
Os	-0.26	-0.44	-0.12	-0.57
Pd	0.43	0.17	0.56	0.23
Pt	0.44	-	-	0.15
Rh	0.03	-0.23	0.09	-0.20
Ru	-0.19	-0.39	-0.03	-0.31
Zn	0.27	0.16	-	0.09

References

1 I. Batatia, P. Benner, Y. Chiang, A. M. Elena, D. P. Kovács, J. Riebesell, X. R. Advincula, M. Asta, M. Avaylon, W. J. Baldwin, F. Berger, N. Bernstein, A. Bhowmik, S. M. Blau, V. Cărare, J. P. Darby, S. De, F. Della Pia, V. L. Deringer, R. Elijošius, Z. El-Machachi, F. Falcioni, E. Fako, A. C. Ferrari, A. Genreith-Schriever, J. George, R. E. A. Goodall, C. P. Grey, P. Grigorev, S. Han, W. Handley, H. H. Heenen, K. Hermansson, C. Holm, J. Jaafar, S. Hofmann, K. S. Jakob, H. Jung, V. Kapil, A. D. Kaplan, N. Karimitari, J. R. Kermode, N. Kroupa, J. Kullgren, M. C. Kuner, D. Kuryla, G. Liepuoniute, J. T. Margraf, I.-B. Magdău, A. Michaelides, J. H. Moore, A. A. Naik, S. P. Niblett, S. W. Norwood, N. O'Neill, C. Ortner, K. A. Persson, K. Reuter, A. S. Rosen, L. L. Schaaf, C. Schran, B. X. Shi, E. Sivonxay, T. K. Stenczel, V. Svahn, C. Sutton, T. D. Swinburne, J. Tilly, C. van der Oord, E. Varga-Umbrich, T. Vegge, M. Vondrák, Y. Wang, W. C. Witt, F. Zills and G. Csányi, *arXiv*, 2024, preprint, arXiv:arXiv:2401.00096, DOI: 10.48550/arXiv.2401.00096.

2 S. Takamoto, C. Shinagawa, D. Motoki, K. Nakago, W. Li, I. Kurata, T. Watanabe, Y. Yayama, H. Iriguchi, Y. Asano, T. Onodera, T. Ishii, T. Kudo, H. Ono, R. Sawada, R. Ishitani, M. Ong, T. Yamaguchi, T. Kataoka, A. Hayashi, N. Charoenphakdee and T. Ibuka, *Nat. Commun.*, 2022, **13**, 2991.

3 S. Grimme, J. Antony, S. Ehrlich and H. Krieg, *J. Chem. Phys.*, 2010, **132**, 154104.

4 Q. Tang, F. Shi, K. Li, W. Ji, J. Leszczynski, A. G. Russell, E. G. Eddings, Z. Shen and M. Fan, *Fuel*, 2020, **280**, 118446.

5 I. Kowalec, L. Kabalan, C. R. A. Catlow and A. J. Logsdail, *Phys. Chem. Chem. Phys.*, 2022, **24**, 9360–9373.

6 L. Kabalan, I. Kowalec, C. R. A. Catlow and A. J. Logsdail, *Phys. Chem. Chem. Phys.*, 2021, **23**, 14649–14661.

7 G. Wang, Y. Morikawa, T. Matsumoto and J. Nakamura, *J. Phys. Chem. B*, 2006, **110**, 9–11.

8 S. Wrobel, in *Principles of Data Mining and Knowledge Discovery*, eds. J. Komorowski and J. Zytkow, Springer, Berlin, Heidelberg, 1997, pp. 78–87.

9 realKD - open-source Java library— Bitbucket, https://bitbucket.org/realKD/realkd/wiki/Home, (accessed 20 November 2025).

10 H. I. Rivera-Arrieta and L. Foppa, *ACS Catal.*, 2025, **15**, 2916–2926.

11 L. Foppa and M. Scheffler, *Digit. Discov.*, 2025, **4**, 2175–2187.
